# Drug-Induced CYP2D6 Phenoconversion Among Individuals with a Psychiatric Disorder: Prevalence and Association with Psychological Distress

**DOI:** 10.3390/pharmaceutics18101319

**Published:** 2026-10-17

**Authors:** Ellen R. Mason, Mohamed Y. Ali, Richéal M. Burns, Priyank Shukla, Aaron Courtenay, David S. Gibson, Elaine K. Murray, Catriona Kelly

**Affiliations:** 1 Personalised Medicine Centre, School of Medicine, Ulster University, C-TRIC Builiding, Altnagelvin Hospital Campus, Glenshane Road, Derry/Londonderry BT47 6SB, UK; mason-e2@ulster.ac.uk (E.R.M.); ali-m36@ulster.ac.uk (M.Y.A.); p.shukla@ulster.ac.uk (P.S.); d.gibson@ulster.ac.uk (D.S.G.); e.murray@ulster.ac.uk (E.K.M.); 2 Department of Social Sciences, Faculty of Science, Atlantic Technological University, F91 YW50 Sligo, Ireland; richeal.burns@atu.ie; 3 Health and Biomedical Research Centre (HEAL), Faculty of Science, Atlantic Technological University, F91 YW50 Sligo, Ireland; 4 School of Pharmacy and Pharmaceutical Sciences, Ulster University, Coleraine BT52 1SA, UK; a.courtenay@ulster.ac.uk

**Keywords:** phenoconversion, pharmacogenomics, CYP2D6, psychiatric disorders, drug–drug–gene interaction, polypharmacy, comorbidity

## Abstract

**Background/Objectives**: Phenoconversion extends pharmacogenomics beyond genetic variation by accounting for non-genetic factors that can also dynamically alter metabolism, influence treatment response, adverse effect risk and overall patient outcomes. CYP2D6 phenoconversion is problematic in people with psychiatric conditions due to the high comorbidity and polypharmacy burden in this population and the significant proportion of psychiatric medications that inhibit CYP2D6 metabolism or are directly metabolised by CYP2D6. Our aim was to estimate the prevalence of CYP2D6 phenoconversion, the association with comorbidity and polypharmacy and the impact on mental health outcomes. **Methods**: Using UK Biobank data, phenoconversion was estimated by applying a standard multiplication factor to genotype-based activity scores according to CYP2D6 inhibitor use, and associations with comorbidity and polypharmacy were assessed. Findings were validated by meta-analysis (PROSPERO, CRD420251177663). Regression analyses assessed the relationship between CYP2D6 phenoconversion and psychological distress, measured using a modified version of the PHQ-4 from UK Biobank. **Results**: Seventeen percent (n = 5942) of UK Biobank participants with a self-reported psychiatric/psychological disorder (n = 35,338) were estimated to be subject to CYP2D6 phenoconversion. CYP2D6 phenoconverted individuals had higher comorbidity and polypharmacy medications, each *p* < 0.001. Twelve studies were included in our meta-analysis, producing a pooled prevalence of 17% (n = 7498, 95% CIs:12–24%, I^2^ = 98%) and 13% (n = 1633, 95% CIs: 11–15%, I^2^ = 24.2%) in a more homogeneous adult-only subgroup. CYP2D6 phenoconversion was significantly associated with higher modified PHQ-4 scores from UK Biobank (*p* < 0.001, r = 0.067), with individuals subject to phenoconversion scoring 10% higher (IRR 1.10, 95% CIs: 1.07–1.13) and having 19% greater odds of higher psychological distress scores (modified PHQ-4 score ≥ 6) (OR 1.19, 95% CIs: 1.11–1.28). **Conclusions**: Overall, CYP2D6 inhibitor use is common in psychiatry, and our findings support the need to consider phenoconversion.

## 1. Introduction

In 2022, just under 16,000 people were waiting for their first mental health appointment in the United Kingdom (UK) [1]. Due to this growing demand for mental health services, pharmacotherapy is often the most accessible option to treat psychiatric disorders. Despite their widespread use, treatment outcomes with psychotropic medications vary considerably, with 20 to 60% of psychiatric patients considered to be treatment-resistant [2]. Consequently, trial-and-error prescribing is commonplace [3]. This prescribing approach is associated with increased medical costs and reduced quality of life [4].

Pharmacogenomics (PGx)-guided prescribing enables clinicians to stratify medication selection and dosing according to a patient’s genetic profile. PGx organisations, including the Clinical Pharmacogenetics Implementation Consortium (CPIC) and the Dutch Pharmacogenetics Working Group (DPWG), have developed evidence-based guidelines for many drug–gene pairs, including psychotropic medications [5,6]. Growing evidence supports this personalised approach to prescribing, with depression having the most established evidence base to date within psychiatry. A recent umbrella review and updated meta-analysis found that depressed patients receiving PGx-guided antidepressant treatment were 41–78% more likely to achieve remission and 20–49% more likely to respond to antidepressant treatment, compared to patients receiving the current standard of care [7]. PGx-guided prescribing in psychiatry is also cost-effective, with 88% of recent studies finding PGx-guided prescribing either cost-effective or cost-saving relative to standard practice [8].

Despite this promise, the clinical utility of PGx-guided prescribing is contingent on the accuracy of genotype-based metaboliser phenotype prediction [9]. While an individual’s genotype remains static, their functional metaboliser phenotype can change in response to non-genetic factors, a process known as phenoconversion [10]. Phenoconversion therefore represents a dynamic, time-varying mismatch between genotype-predicted and functional drug-metabolising capacity. This distinction is clinically important because a PGx test provides static genetic information at a single time point, whereas subsequent changes in functional metaboliser status may alter the appropriateness of genotype-guided medication selection or dosing. Phenoconversion is therefore a clinically relevant extension of PGx that may contribute to the occurrence of drug–drug–gene interactions (DDGIs) when changes in metaboliser phenotype status affect the metabolism of a relevant medication.

A key driver of phenoconversion is the use of concomitant medications, which can induce or inhibit the cytochrome P450 (CYP450) enzymes that are involved in drug metabolism [10]. Psychotropic polypharmacy, previously defined as the concurrent use of two or more psychotropic medications, is highly prevalent in psychiatric populations, with reported rates ranging from 13% to 90%, depending on the clinical setting, diagnosis, and patient demographics [11,12]. Furthermore, those with severe mental illnesses also have a higher prevalence of non-psychiatric comorbidities [13]. Of particular relevance is CYP2D6, which is involved in the metabolism of a considerable number of common medications, including psychotropic medications and is also susceptible to inhibition by various drugs, including paroxetine, fluoxetine and bupropion [14]. This combination of a high prevalence of polypharmacy, comorbidity and widespread use of CYP2D6 inhibitors in psychiatry may place these individuals at an increased risk of phenoconversion, and consequently at risk of adverse outcomes.

While the prevalence of phenoconversion is an important metric, its clinical significance lies in its potential to modify treatment response, adverse effect risk and overall patient outcomes in routine care. Therefore, our aims were to (1) estimate the prevalence of CYP2D6 phenoconversion among individuals with a psychiatric disorder and its relationship with comorbidity and polypharmacy and (2) establish the relationship between CYP2D6 phenoconversion status and mental health outcomes using a modified version of the Patient Health Questionnaire-4 (PHQ-4) from UK Biobank. In doing so, this study moves beyond prevalence estimation to examine whether CYP2D6 phenoconversion is associated with a modified psychological distress score in a large real-world cohort.

## 2. Materials and Methods

### 2.1. Estimation of CYP2D6 Phenoconversion in Participants from the UK Biobank

The prevalence of CYP2D6 phenoconversion was first estimated in a cross-sectional analysis of a cohort of individuals with a self-reported psychiatric/psychological problem or disorder from UK Biobank. At the initial assessment visit (Instance 0, 2006–2010), participants self-reported serious medical conditions or disabilities using a system based on the International Classification of Diseases, 10^th^ Revision (ICD-10). Any participants reporting any condition within the “psychological/psychiatric problem” category were included in our analysis (full list of conditions included in Supplementary Methods S1). Comorbidity burden and polypharmacy were quantified using the total number of self-reported conditions/disabilities from the same ICD-10-based system and medications recorded at Instance 0, inclusive of psychiatric/psychological conditions and psychotropic medications, respectively. Medication use was reported during a verbal interview with a trained nurse and coded using the UK Biobank treatment coding list (Field 20003, Data-Coding 4).

CYP2D6 genotype calls were obtained directly from UK Biobank whole-genome sequencing data (Field 24065), which had been processed using DRAGEN 3.7.8 (Illumina, Inc., San Diego, CA, USA), and were extracted via the UK Biobank Research Analysis Platform (UKB-RAP). However, it should be noted the DRAGEN CYP2D6 caller identifies a predefined set of star alleles and structural variants; therefore, some rare or structural alleles may not be fully identified [15]. Metaboliser phenotypes and activity scores were assigned using the ‘Gene-specific Information Tables for CYP2D6’ on ClinPGx (clinpgx.org), including the CYP2D6 Diplotype-Phenotype Table, CYP2D6 Allele Functionality Table and the CYP2D6 Phenotype Table (tables accessed in September 2025). Participants with multiple possible CYP2D6 genotypes were only included when each possible genotype resulted in the same metaboliser phenotype. To estimate the prevalence of CYP2D6 phenoconversion among individuals with a psychiatric/psychological disorder, we created a subset of participants who had both a CYP2D6 genotype call and a reported psychiatric/psychological disorder at Instance 0. Those with an indeterminate phenotype were subsequently excluded, as phenoconversion status could not be determined.

To estimate CYP2D6 phenoconversion, we used the tutorial by Cicali and colleagues on integrating CYP2D6 phenoconversion into clinical pharmacogenetics [16]. Accordingly, if a participant was on a strong CYP2D6 inhibitor (e.g., fluoxetine, paroxetine) their activity score was multiped by zero, resulting in poor metaboliser status, whereas if a participant was on a moderate CYP2D6 inhibitor (e.g., duloxetine, doxepin), their activity score was multiplied by 0.5, and they may or may not have converted to another metaboliser phenotype. If a participant was on both a strong and a moderate inhibitor, the multiplication factor for the strong inhibitor (0) was used. To identify those at risk of CYP2D6 phenoconversion, we used their self-reported medications at Instance 0, identified individuals in the cohort who reported that they were taking a strong or moderate inhibitor of CYP2D6 and applied the multiplication factor. Individuals were considered to have phenoconverted if their genotype-predicted phenotype differed from their inhibitor-adjusted phenotype. We used the ‘Drug Interactions Flockhart Table’ as the source of CYP2D6 inhibitors, consistent with the approach of Borges and colleagues (list of inhibitors in Supplementary Methods S2) [14]. We also used Drugs.com (www.drugs.com) and DrugBank (www.drugbank.com) to search for any brand names for the CYP2D6 inhibitors to ensure a comprehensive search. The distributions of genotype-predicted and estimated phenoconversion-corrected CYP2D6 metaboliser phenotypes were compared using the Stuart–Maxwell test of marginal homogeneity, accounting for paired categorial nature of the data.

### 2.2. Meta-Analysis of Rates of CYP2D6 Phenoconversion in the Existing Literature

To validate CYP2D6 phenoconversion prevalence data obtained from UK Biobank, a systematic review and meta-analysis of the existing literature were performed. The protocol for this systematic review and meta-analysis was registered with PROSPERO (CRD420251177663, Supplementary Methods S3) on 03/10/2025, and all data were extracted between 09/02/2026 and 16/02/2026. PRISMA 2020 and MOOSE guidelines (Tables S1 and S2) were followed for this review. Embase, MEDLINE and PsycINFO were searched from inception to 3 November 2025, using Ovid 2026, with reference lists checked for additional studies. To maximise our search, we used Ovid’s explosion feature, wildcard operators (*) and Boolean operators (AND/OR) to combine concepts. Studies were considered eligible if they (1) had a study cohort with any psychiatric disorder covered by the International Classification of Diseases, Tenth Revision (ICD-10), Chapter V, and (2) reported the prevalence of CYP2D6 phenoconversion as either a direct number/proportion of individuals who phenoconverted or reported CYP2D6 metaboliser phenotype distributions before and after phenoconversion, from which prevalence could be estimated using the net change. Eligible studies included any observational study design. We excluded any study that did not meet these inclusion criteria or was not peer-reviewed, as such studies may limit the quality of the methodology, and any studies that were not in the English language due to practical limitations in translating the full-text. We also excluded studies that only reported the number/proportion of individuals in the cohort prescribed CYP2D6 inhibitors, as they could include poor metabolisers who would not undergo phenoconversion. All relevant data was available in the published manuscript or Supplementary Materials.

Study screening using Covidence (Veritas Health Innovation, Melbourne, Australia; https://www.covidence.org) and quality appraisal using the critical appraisal checklist for studies reporting prevalence by the Joanna Briggs Institute (JBI), were each carried out by two independent reviewers (ERM and MYA), and any disagreements were resolved through discussion. Data extraction was carried out by one reviewer (ERM) and checked by another (MYA). Results are presented in tables for study characteristics, methods and prevalence findings and in forest plots for the meta-analysis outputs. Assessment of confounding was not applicable to this review, as included studies reported the prevalence in a single cohort rather than case-control comparisons. The certainty of evidence was assessed narratively using the GRADE framework, as there is currently no specific GRADE guidance for prevalence studies.

To conduct a meta-analysis of prevalence, we followed the methodology of Barendregt and colleagues [17]. Where there was more than one study by the same author using the same study cohort, the study with the largest sample size was retained to avoid duplication of participants. Meta-analyses were conducted using R (version 4.5.2). As described in the methods by Barendregt and colleagues, we transformed our prevalence values using the logit transformation to address the bounded nature of proportions and to stabilise the variance. We used a random-effects model with inverse-pooling and restricted maximum likelihood (REML) estimation of between-study variance [17]. Results were presented with 95% confidence intervals (CIs) and prediction intervals (PIs) and visualised using forest plots. Heterogeneity was assessed using various measures, including the I^2^ statistic, and publication bias was explored using the Luis Furuya-Kanamori (LFK) index, Doi plots, and Egger’s regression tests when there were ten or more studies [18,19]. To reduce methodological heterogeneity, a post hoc decision was made to exclude two studies using a metabolic-ratio-based method to determine CYP2D6 phenoconversion, and two studies that considered only strong inhibitors were excluded from the meta-analysis, as these were considered methodologically distinct from the remaining studies.

### 2.3. Association of Potential CYP2D6 Phenoconversion and Psychological Distress in Participants from UK Biobank

To assess the relationship between potential CYP2D6 phenoconversion status and patient-reported psychological distress, we used a modified version of the PHQ-4 using UK Biobank fields 2050, 2060, 2070 and 2080 at Instance 0. These items assessed depressed mood, unenthusiasm/disinterest, tenseness/restlessness and tiredness/lethargy over the previous two weeks. Participants who responded “Do not know” or “Prefer not to answer” to any of the four questions were excluded. These four items were selected to provide a measure of psychological distress that was collected at the same time as the medication data used to estimate phenoconversion. The PHQ-4 is a validated four-item measure of psychological distress, combining the PHQ-2 (depression) and the Generalised Anxiety Disorder 2-item scale (GAD-2)(anxiety), with a score ranging from zero to twelve [20]. The first two items (2050 and 2060) available from UK Biobank correspond closely to the PHQ-2; however, the latter two (2070 and 2080), are modifications of the GAD-2 items of the validated PHQ-4. Therefore, the resulting score was treated as a modified version of the PHQ-4 (PHQ-4 questions and UK Biobank fields used are detailed in Supplementary Methods S4).

We first excluded any participants who had missing covariate data. Modified PHQ-4 scores and CYP2D6 phenoconversion status were compared using the Mann–Whitney U test given the non-normal distribution of scores, and the effect size was quantified using the rank-biserial correlation coefficient. Given that the modified PHQ-4 scores from UK Biobank were a bounded count outcome and exhibited overdispersion (mean = 3.72, variance = 10.00), negative binomial regression was used, with incidence rate ratios (IRRs) as the outcome with 95% CIs. Binary logistic regression was also used as an exploratory analysis to evaluate whether CYP2D6 phenoconverted individuals were more likely to have higher modified PHQ-4 scores (≥6). This threshold was selected given the similarity between the questions provided by UK Biobank and those of the validated PHQ-4, for which scores ≥ 6 indicate moderate (6–8) and severe (9–12) psychological distress. Furthermore, increasing PHQ-4 scores have been strongly associated with functional impairment warranting further assessment [20]. However, as the modified measure used in this analysis has not been validated, this threshold was used solely as an exploratory indicator of higher scores. Results of this analysis are reported as odds ratios (ORs) with 95% CIs. Both models accounted for genotypic phenotype, age, sex, ethnicity, Townsend deprivation index, number of self-reported medications, number of self-reported indications, smoking status and alcohol use. For regression analyses, ethnicity was dichotomised as ‘British or Irish’ vs. ‘Other’ due to insufficient numbers in individual ethnic minority groups.

## 3. Results

### 3.1. Seventeen Percent of Patients with a Psychiatric/Psychological Problem in the UK Biobank Liable to CYP2D6 Phenoconversion

In the UK Biobank, there were a total of 490,278 individuals with a CYP2D6 genotype call to extract. While most of these participants had an identified genotype, 3591 participants had no call for their genotype and so were removed from the dataset. Some participants also had more than one possible genotype (4927) and so the phenotype was identified for each possible genotype, and if they did not match, the participant was removed from the dataset (25 were removed). There were also 189 participants who subsequently decided to withdraw their data from UK Biobank, leaving a final cohort of 486,473 participants for whom we could identify their CYP2D6 metaboliser phenotype. In this cohort, 33,817 (6.95%) participants were poor CYP2D6 metabolisers, 191,969 (39.46%) were intermediate metabolisers, 241,074 (49.56%) were normal metabolisers, 8218 (1.69%) were ultra-rapid metabolisers, and 11,395 (2.34%) were participants who were indeterminate due to the presence of an allele with an unknown or uncertain function.

After removal of participants with an indeterminate CYP2D6 phenotype, there were 35,338 participants who also reported a psychiatric/psychological problem at Instance 0. Self-reported conditions are detailed in Table S6. The metaboliser phenotype distribution within this cohort was very similar to that of the total cohort, with 2373 (6.72%) participants who were poor CYP2D6 metabolisers, 14,406 (40.77%) who were intermediate metabolisers, 17,958 (50.82%) who were normal metabolisers, and 601 (1.70%) who were ultra-rapid metabolisers.

**Figure 1:**
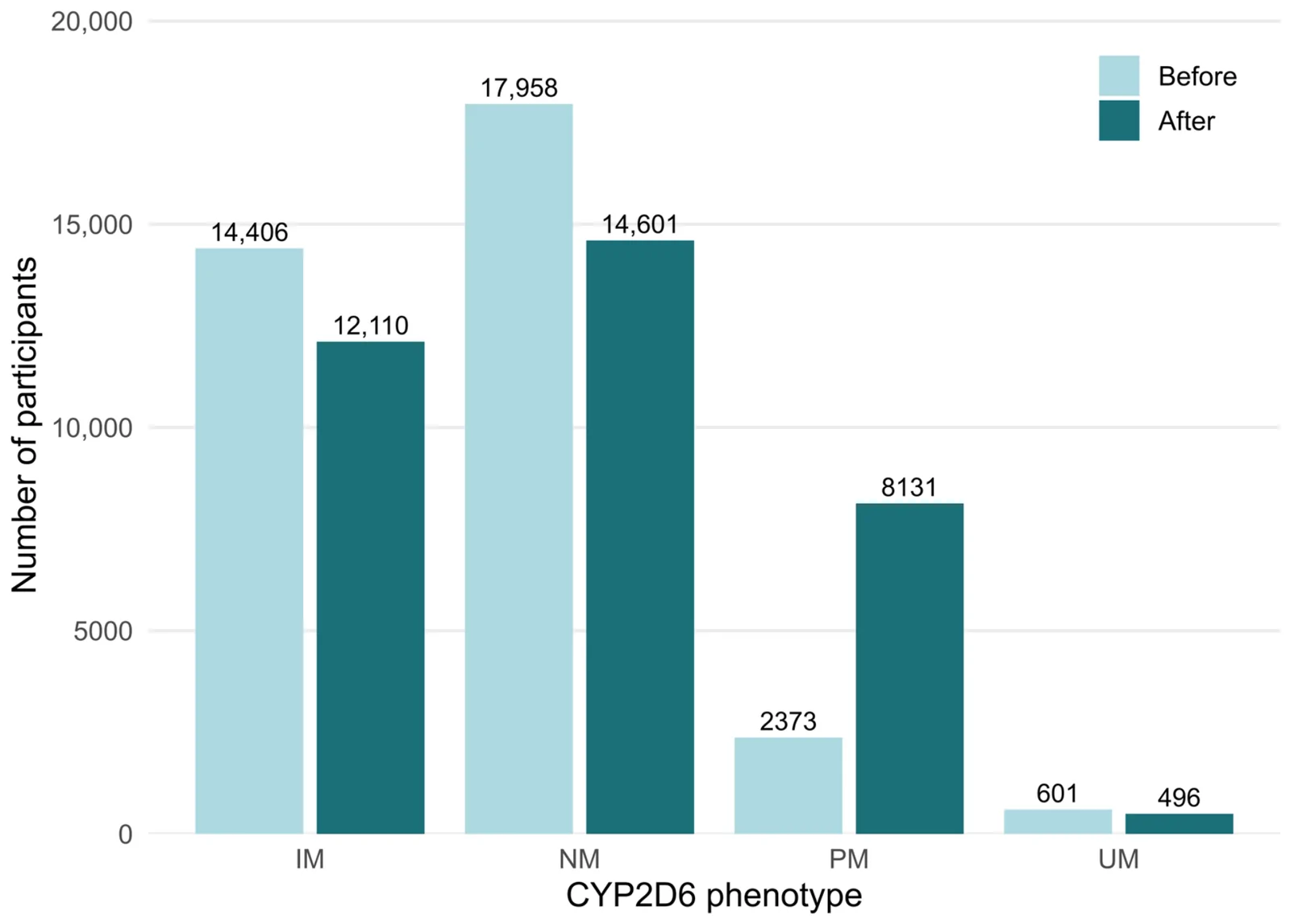
CYP2D6 phenotype distribution before and after estimated phenoconversion among individuals with a psychiatric/psychological disorder in the UK Biobank. Bar chart showing the number of individuals classified into each CYP2D6 metaboliser phenotype before (pale blue) and after (dark teal) phenoconversion. IM, intermediate metaboliser; NM, normal metaboliser; PM poor metaboliser, UM, ultrarapid metaboliser.

A total of 6508 (18.42%) participants were prescribed at least one CYP2D6 inhibitor, with 6155 (17.42%) on at least one strong inhibitor and 353 (1.00%) people on at least one moderate inhibitor. Application of the estimated phenoconversion correction algoritm resulted in a significant change in the distribution of CYP2D6 metaboliser phenotypes (Stuart–Maxwell test, χ^2^(3) = 5777.8, *p* < 0.001). Overall, 5942 (16.81%) participants were estimated to have a change in CYP2D6 phenotype classification. This excluded the 397 participants on a strong CYP2D6 inhibitor who were already poor metabolisers and the 169 participants on a moderate inhibitor for whom the standard multiplication factor did not change their phenotype. The majority of estimated phenotype reclassifications were from normal to poor metabolisers (n = 3187, 53.6%) or intermediate to poor metabolisers (n = 2473, 41.6%), which together accounted for 95.3% of all estimated reclassifications. The changes in phenotype distribution are shown in Figure 1, with individual phenotype transitions detailed in Table 1.

The most commonly prescribed inhibitors were fluoxetine (n = 4895) and paroxetine (n = 1254) (Table S7). Individuals at risk of CYP2D6 phenoconversion were more likely to be female (69.7% vs. 63.2%), had higher levels of co-morbidities (3.70 [SD 2.39] vs. 3.55 [SD 2.32]), more medications (5.76 [SD 3.39] vs. 4.83 [SD 3.24]), and had a higher prevalence of current or previous smoking (17.7% and 36.5% vs. 16.9% and 33.9%, respectively), all *p* < 0.001 (Table S8).

Values are represented as n (%), with percentages calculated within each genotype-predicted phenotype (row percentages). Rows represent genotype-predicted CYP2D6 phenotypes, and columns represent phenoconversion-corrected phenotypes. Diagonal cells indicate concordance between genotype-predicted and phenoconversion-corrected CYP2D6 phenotypes, while off-diagonal cells indicate phenotype reclassification following drug-induced phenoconversion.

### 3.2. Meta-Analysis Confirms CYP2D6 Phenoconversion Rates of 13–17%

To validate our findings using a meta-analysis, we carried out a literature search, identifying 818 articles (553 from Embase, 136 from MEDLINE and 129 from PsycINFO; Figure S1). Reference checking did not identify any additional studies. After 232 duplicates were removed, the titles and abstracts of the remaining 586 studies were screened. Thirty studies then underwent full-text evaluation. Of these, 18 met the inclusion criteria and were included in the review, and 12 were included in the meta-analysis [21,22,23,24,25,26,27,28,29,30,31,32,33,34,35,36,37,38]. Excluded studies and the reasons for their exclusion are provided in Table S9 [39,40,41,42,43,44,45,46,47,48,49]. Study characteristics and extracted phenoconversion data can be found in Table 2; all other data extraction tables and narrative synthesis results are provided in the Supplementary Materials.

**Figure 2:**
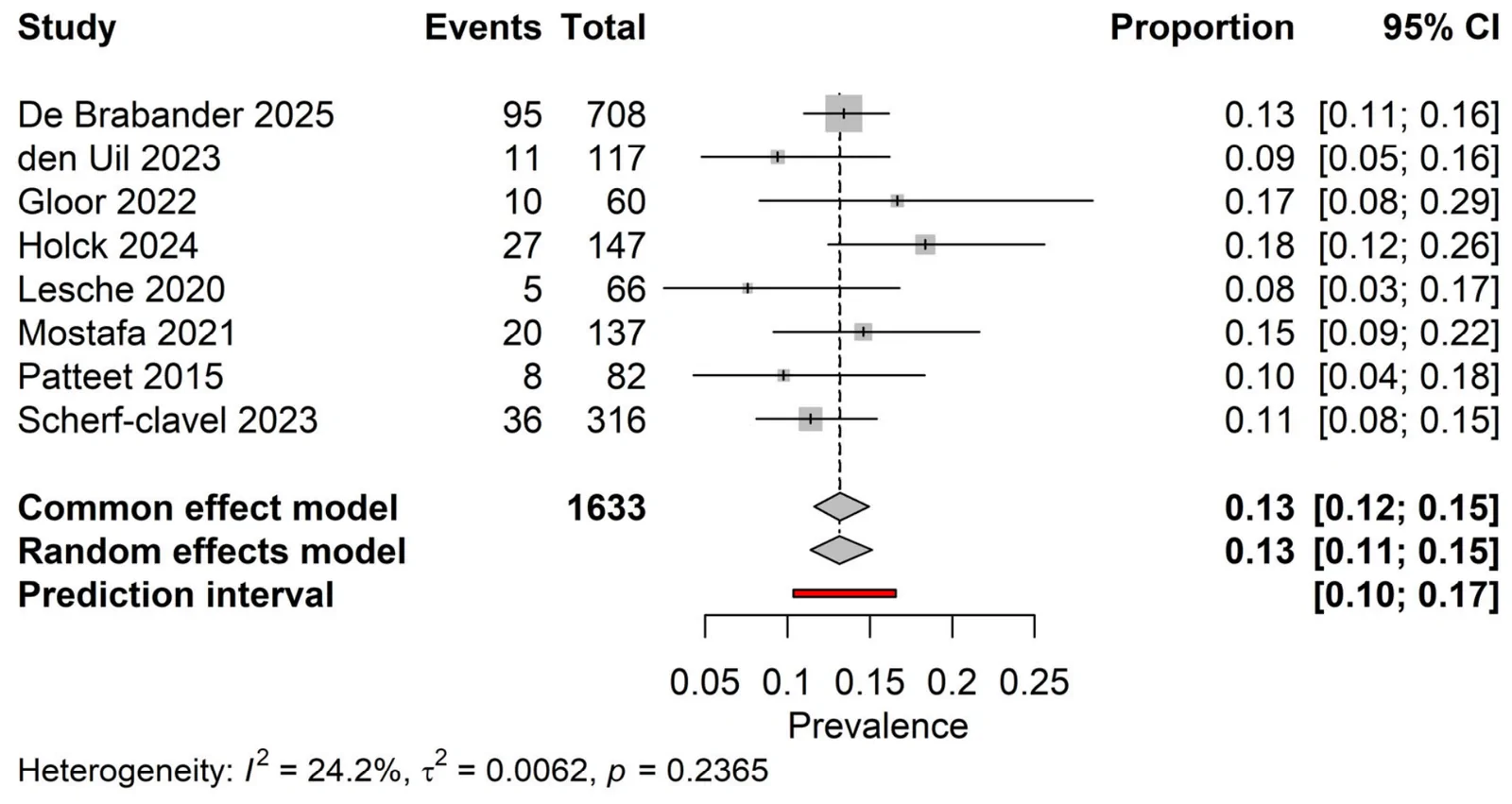
Subgroup meta-analysis of CYP2D6 phenoconversion prevalence among adults with a psychiatric disorder using the logit transformation. Forest plot of the pooled prevalence of CYP2D6 phenoconversion among adults (≥19 years old) with a psychiatric disorder using a random-effects model with the logit transformation. Squares represent individual study estimates weighted by study size, with the horizontal line representing the 95% confidence intervals (CIs), while the diamond represents the pooled prevalence and the 95% CIs. The red horizontal bar represents the prediction interval. Heterogeneity was assessed using the I^2^ statistic (I^2^ = 24.2%) [21,23,26,28,32,33,34,37].

Our initial meta-analysis of 12 studies, using the logit transformation and a random-effects model, demonstrated substantial heterogeneity (I^2^ = 98%), with a pooled prevalence of 17% (95% CIs: 12–24%, PIs: 4–50%) (Figure S2). Sensitivity analysis using the Freeman–Tukey double arcsine transformation produced similar estimates and is reported in Figure S3. Egger’s test (t = −3.62, df = 10, *p* = 0.0047, bias = −7.07, SE = 1.96), the Doi plot and the Galbraith plot all suggested major asymmetry (Figures S4 and S5). The leave-one-out sensitivity analysis indicated that omitting the study by Patel et al. [35], the largest study, reduced heterogeneity (I^2^) from 98% to 93% (Table S14). A subgroup analysis by age (Figure S6), identified the exclusively adult subgroup (n = 8) as a more homogeneous group of studies (I^2^ = 24.2%), with a pooled prevalence of 13% (95% CIs: 11–15%, PIs: 10–17%) (Figure 2) (sensitivity analysis using Freeman–Tukey transformation in Figure S7). Doi and Galbraith plots for this adult subgroup showed minor asymmetry (LFK index = −1.79) (Figures S8 and S9). A meta-regression was not performed due to the small number of studies.

The certainty of evidence was assessed narratively using the GRADE framework, with the 18 observational studies beginning at low certainty by default. No downgrading was applied for risk of bias, although some concerns were identified regarding variation in the comprehensiveness of CYP2D6 genotyping. Based on the information reported, assessment of copy number variants and CYP2D6/CYP2D7 hybrid alleles varied across studies, with some studies appearing not to assess these structural variants comprehensively. This may have resulted in misclassification of genotype-predicted phenotype and consequently influenced estimates of phenoconversion prevalence. The level of certainty was, however, downgraded for inconsistency as substantial heterogeneity was observed in the overall meta-analysis (I^2^ = 98%), which was only partially explained by the age-group-based subgroup analysis (adult-only analysis, I^2^ = 24.2%). No downgrading was applied for indirectness as all studies were conducted in psychiatric populations, using standardised genotype-to-phenotype translation methods, directly relevant to the review question. Imprecision was also not considered a major concern as most studies (67%) met the pre-specified minimum sample size threshold (Supplementary Results). Although the 95% confidence interval around the overall pooled prevalence estimate was relatively wide (12–24%), both bounds indicate that CYP2D6 phenoconversion potentially affects a substantial proportion of psychiatric patients; however, the magnitude of this prevalence should be interpreted with caution. However, there was a suggestion of publication bias in the overall meta-analysis, indicated by the results of Egger’s test, the Doi plot (LFK index = −10.39) and the Galbraith plot. Major asymmetry persisted following the removal of the largest study by Patel et al. [35] (LFK index = −3.2) (Figure S10); however, the adult-only analysis demonstrated only minor asymmetry (LFK index = −1.79). Overall, the certainty of evidence was rated as very low, downgraded from low for inconsistency and publication bias.

### 3.3. Estimated CYP2D6 Drug-Induced Phenoconversion Is Associated with Higher Levels of Psychological Distress in UK Biobank

Of the 35,338 participants in the UK Biobank with both a CYP2D6 genotype call and a psychiatric/psychological disorder, 30,824 participants answered the four psychological distress items and had complete covariate data, and so were included in the psychological distress analysis. CYP2D6 phenoconverted individuals had a higher mean modified PHQ-4 score than non-phenoconverted individuals (4.14 vs. 3.63, *p* < 0.001), and a higher proportion had a modified PHQ-4 score ≥ 6 (27.6% vs. 23.0%, *p* < 0.001) (Table S16). Comparison of modified PHQ-4 score distributions using the Mann–Whitney U test confirmed this difference (W = 59,864,139, *p* < 0.001); however, the effect size was small (rank-biserial r = 0.067). Negative binomial regression showed that those at risk of CYP2D6 phenoconversion had 10% higher modified PHQ-4 scores after accounting for covariates (IRR 1.10, 95% CI 1.07–1.13, *p* = 8.48 × 10^−14^) (Table S17). Binary logistic regression was consistent with the negative binomial regression results, with those at risk of CYP2D6 phenoconversion having 19% higher odds of modified PHQ-4 scores ≥ 6 (OR 1.19, 95% CI 1.11–1.28, *p* = 1.85 × 10^−6^) (Table 3).

## 4. Discussion

Our findings show that CYP2D6 inhibitor use is common among individuals with a psychiatric/psychological disorder, with ~17% of individuals in the UK Biobank cohort at risk of CYP2D6 phenoconversion. Individuals who were subject to phenoconversion were more likely to be female and carry both a greater medication and comorbidity burden, consistent with the DDGIs that drive CYP2D6 phenoconversion [21]. Phenoconversion risk in the UK Biobank cohort was primarily due to the common prescribing of the potent CYP2D6 inhibitors fluoxetine and paroxetine, which together made up 94% of CYP2D6 inhibitor prescriptions.

Fluoxetine and paroxetine are both selective serotonin reuptake inhibitors (SSRIs). Despite downward trends in prescribing in the UK, they both remain among the top 10 most commonly prescribed antidepressants in the UK, with 7.5 million and 1.3 million items prescribed, respectively, in 2024/25 [50,51]. SSRIs are commonly prescribed and recommended by NICE clinical guidelines as they are generally considered to be effective, tolerable and safe pharmacological treatments [52]. However, because both of these SSRIs are also potent CYP2D6 inhibitors, their continued widespread use may contribute to the occurrence of phenoconversion among individuals prescribed these two medications [14]. Although alternative antidepressants with reduced CYP2D6 inhibitory activity are available, the frequency at which fluoxetine and paroxetine are prescribed highlights the potential relevance of considering phenoconversion in clinical practice within psychiatry.

Fluoxetine and paroxetine each inhibit the CYP2D6 enzyme through different mechanisms. Fluoxetine is thought to inhibit CYP2D6 primarily through competitive inhibition, while paroxetine has been found to quasi-irreversibly inhibit CYP2D6 through mechanism-based inhibition [53,54]. However, fluoxetine and its active metabolite, norfluoxetine, also have prolonged half-lives of 1–4 and 7–15 days, respectively, resulting in sustained CYP2D6 inhibition that persists well beyond discontinuation of fluoxetine [55]. These half-lives can also increase with chronic fluoxetine use, further prolonging this inhibition [56]. The clinical consequences of prolonged inhibition can be significant and can be particularly problematic given that CYP2D6 is involved in the metabolism of 20–25% of all drugs, including fluoxetine and paroxetine themselves, increasing the risk of CYP2D6 substrate accumulation to potentially toxic plasma concentrations [57]. Another strong CYP2D6 inhibitor that can be commonly prescribed in psychiatry, as seen in the studies in our review, is bupropion. However, in the UK, bupropion is currently only prescribed for smoking cessation, likely explaining why it was not commonly prescribed in the UK Biobank cohort [58].

Other commonly prescribed psychotropic medications, such as sertraline and duloxetine, can also inhibit CYP2D6; however they are classified as weak/moderate CYP2D6 inhibitors and therefore will not influence CYP2D6 metabolism to the same extent [14,50]. Common prescribing of strong and moderate CYP2D6 inhibitors within psychiatry puts these individuals at increased risk of DDGIs and potentially serious adverse effects.

There is published evidence of serious adverse effects that have resulted from DDGIs following CYP2D6 phenoconversion. One review discussed how the co-administration of strong CYP2D6 inhibitors alongside the beta-blocker metoprolol, can increase metoprolol levels by 8-fold, resulting in severe adverse events such as bradycardia and hypotension [59]. Additionally, two case reports reported individuals experiencing delirium and excessive sedation as a result of DDGIs involving either fluoxetine or paroxetine, driven by CYP2D6 phenoconversion from an intermediate to a poor metaboliser in each report [60,61]. Not surprisingly, one study found that young adults with a CYP2D6 poor metaboliser phenotype, who were also on fluoxetine, had an increased risk of emergency department contacts [62]. Importantly, the clinical relevance of these interactions extends beyond psychiatric care. Individuals with psychiatric disorders frequently have multimorbidity and may receive CYP2D6 substrates for non-psychiatric conditions alongside psychotropic CYP2D6 inhibitors [13]. This is particularly relevant in primary care, where multimorbidity and polypharmacy are commonly managed and medication regimens may span multiple clinical specialities. As the study cohort was identified within a general population biobank rather than a specialist mental health setting, our findings may have broader relevance to these prescribing contexts. Consideration of phenoconversion during routine medication reviews could help identify clinically important DDGIs and inform prescribing and deprescribing decisions as medication regimens change over time.

Our findings show that there is an association between estimated drug-induced CYP2D6 phenoconversion status and the modified version of the PHQ-4 used in the UK Biobank. In addition to affecting quality of life, evidence also suggests that there is an association between economic burden and the severity of depression and anxiety. Popkov and colleagues found in their 2025 US study that increased severity of depression was associated with up to 48% higher healthcare costs, while improvement only reduced costs by up to 15% [63]. Kaavelaars and colleagues reported similar findings for anxiety in the US in 2023, demonstrating that both direct and indirect costs escalated with symptom severity [64]. Although healthcare utilisation and costs were not assessed in the present study, these findings provide a rationale for future research investigating whether CYP2D6 phenoconversion and its potential clinical consequences are associated with economic outcomes. While PGx-guided prescribing in psychiatry is cost-effective and likely cost-saving, this approach currently rarely considers concomitant medications [8]. By integrating phenoconversion risk into person-centred medication reviews of multimorbidity and polypharmacy, which may be guided by PGx testing, both clinical and economic outcomes could be further optimised beyond what PGx-guided prescribing alone can achieve.

The dynamic nature of phenoconversion is something that can complicate its integration into clinical practice. A person’s functional CYP2D6 phenotype can shift with every change to their medication list, demanding a responsive solution, unlike a static PGx report. Current electronic health record systems largely do not consider phenoconversion. However, Tabula Rasa HealthCare has developed a patented risk score that works within the clinical decision support system MedWise to account for both pharmacokinetic and pharmacodynamic factors that can cause medication-related problems, such as drug-induced phenoconversion [65]. Various case reports have demonstrated the risk score’s capability to allow pharmacists to identify CYP2D6 phenoconversion [61]. Web-based phenoconversion calculators have also been developed to allow clinicians to input a patient’s genotype and medication list to receive an estimate of the individual’s actual metabolising capability, though these remain separate from prescribing workflows [16]. Despite the development of such algorithms and tools to aid its integration, phenoconversion currently remains largely absent from routine prescribing decisions and still relies heavily on the pharmacist’s knowledge of interpreting a PGx report, limiting their real-world clinical application [66].

Our UK Biobank analysis, systematic review and meta-analysis had several limitations. For the UK Biobank analysis, the medications and psychiatric/psychological problems at Instance 0 were self-reported and therefore may not have been completely accurate. In particular, the use of the broad self-reported definition of psychiatric/psychological problems may not reflect clinically confirmed psychiatric diagnoses. Information on the dose, adherence, and duration of CYP2D6 inhibitor use was also unavailable, which may have influenced the extent of CYP2D6 inhibition. However, using data at Instance 0 allowed us to maximise the cohort, as this was the time point at which most participants completed the questionnaires. As this data was also cross-sectional, phenoconversion was algorithmically estimated based on concomitant CYP2D6 inhibitor use rather than directly measured using a metabolic ratio. Although this approach is consistent with CPIC-endorsed methods and is accepted as an appropriate estimation of CYP2D6 phenoconversion, it may have resulted in either underestimation or overestimation of the observed phenoconversion prevalence, which we sought to address with a meta-analysis of the current literature [16]. Given the complexity of CYP2D6, the predefined set of star alleles and structural variants used by DRAGEN may also have contributed to potential genotype-predicted phenotype misclassification. The observed association between CYP2D6 phenoconversion and psychological distress should also be interpreted with caution, as fluoxetine and paroxetine accounted for most CYP2D6 inhibitor exposure, and their use may reflect differences in underlying psychiatric illness/severity, potentially confounding this relationship. Our use of a modified PHQ-4 score from the UK Biobank is also a limitation. Although the selected UK Biobank fields captured symptoms of depression and psychological distress, the measure differs from the validated PHQ-4 and has not itself been validated. Therefore, the psychological distress findings should be interpreted with caution and require replication using a validated measure. Finally, because the studies included in our meta-analysis were not typical prevalence studies with large sample sizes, the prevalence estimates should be interpreted with caution. The substantial heterogeneity observed (I^2^ = 98%) was only partially explained by age-group subgroup analysis, suggesting that other factors may influence the prevalence of CYP2D6 phenoconversion. However, the prevalence observed in our larger UK Biobank analysis was consistent with the findings of our meta-analysis, supporting its validity.

## 5. Conclusions

To our knowledge, this is the largest assessment of both drug-induced CYP2D6 phenoconversion prevalence in psychiatry and its association with psychological distress to date. Our findings support the need to consider inhibitor-mediated modification of CYP2D6 phenotypes and justify prospective studies evaluating its clinical consequences. Although phenoconversion was associated with psychological distress in this cohort, further research is required to replicate this association and better understand its relationship with mental health outcomes. Prospective longitudinal studies incorporating direct measures of CYP2D6 activity, such as those obtained using metabolic ratios, could help to establish the clinical consequences of drug-induced CYP2D6 phenoconversion in psychiatry. The findings of this study highlight the future need to consider phenoconversion as part of PGx guidelines. Yet, there is significant work to be undertaken in standardising how phenoconversion is estimated, especially for pharmacogenes beyond CYP2D6. The development of clinical decision software that can incorporate drug-induced phenoconversion in real time is also required to ensure patient safety.

## Figures and Tables

**Table 1:** Cross-tabulation of genotype-predicted and estimated phenoconversion-corrected CYP2D6 phenotypes.

Genotype-Predicted Phenotype	Corrected PM	Corrected IM	Corrected NM	Corrected UM	Total
PM	2373 (100%)	0 (0%)	0 (0%)	0 (0%)	2373
IM	2473 (17.2%)	11,933 (82.8%)	0 (0%)	0 (0%)	14,406
NM	3187 (17.7%)	177 (1.0%)	14,594 (81.3%)	0 (0%)	17,958
UM	98 (16.3%)	0 (0%)	7 (1.2%)	496 (82.5%)	601

PM, poor metaboliser; IM, intermediate metaboliser; NM, normal metaboliser and UM, ultrarapid metaboliser.

**Table 2:** Study characteristics. Descriptive characteristics of included studies that reported CYP2D6 phenoconversion among individuals with a psychiatric disorder.

Author (Year)	Sample Size	Country	Race, Ethnicity or Genetic Ancestry ^3^	Age	Sex Distribution	Study Design	Setting	Medication-Based Inclusion Criteria	Diagnoses
Preskorn et al. [36] (2013)	900	United States	White 85% Black or African American 12% Other 1% American Indian or Alaska Native <1% Asian < 1%	Mean: 44.7 SD: ±14.5 Range: 18–92	Female: 61% Male: 39%	Cross-sectional	Outpatient clinic	Receiving outpatient treatment with venlafaxine extended release	-
Patteet et al. [34] (2015)	82	Belgium	-	Range: 19–66	Female: 36.6% Male: 63.4%	Cross-sectional	Hospital-based	Patients who were treated with aripiprazole, haloperidol, risperidone, paliperidone, and/or zuclopenthixol	-
Lesche et al. [32] (2020)	66	Australia	Northern and western Europe 85%	Mean: 40 SD: ±10	Female: 27% Male: 73%	Cross-sectional	In- and out-patient clinics	Prescribed and taking clozapine	-
Hole et al. [29] (2021)	59 on 150 mg/d BUP and 34 on ≥300 mg/d BUP	Norway	-	Median: 49 Range: 24–75	Female: 64% Male: 36%	Cross-sectional	Hospital-based	Venlafaxine ± bupropion	-
Mostafa et al. [33] (2021)	137	Australia	Caucasian 89% Asian 4% Not recorded 3% African 2% Middle Eastern 1%	Mean: 78.5 Range: 60–97	Female: 61% Male: 39%	Cross-sectional	Acute and residential in-patients	-	-
Jallaq et al. [30] (2021)	277	United States	White 83.8% Black 10.8% Other 5.4%	Mean: 14.3 SD: ±2.4 Range: 6.0–19.6	Female: 65.7% Male: 34.3%	Cohort	Hospital outpatient clinic	Prescription of aripiprazole	MDD 65.0% BD 28.9% DMDD 5.8% Not specified 0.36%
Gloor et al. [26] (2022)	60	Switzerland	The study population was assumed to be mainly of European descent	Median: 47 Range: 22–65	Female: 61.7% Male: 38.3%	Cross-sectional	Psychiatric outpatient clinic	Under stable antidepressant treatment for at least 6 weeks	-
den Uil et al. [23] (2023)	117	Netherlands	-	Mean: 42.5 SD: ±11.8	Female: 60.7% Male: 39.3%	Cohort	“Body and Life” outpatient psychiatric clinic	-	SCZ 36.8% BD: 24.8% DEP: 11.1% PD: 9.4% Other 9.0% NDD: 6.8% PTSD: 5.1% ANX: 1.7% ED 1.7% SSD: 0.9% Addiction: 0.9%
Scherf-Clavel et al. [37] (2023)	316	Germany	-	Mean: 44.2 SD: ±15.4 Range: 18–84	Female: 54.1% Male: 45.9%	Cross-sectional	University hospital inpatient clinics	Patients who received patients who received venlafaxine, amitriptyline, mirtazapine, sertraline, escitalopram, risperidone and quetiapine	-
Tonti et al. [38] (2024)	193	United States	Caucasian 78.2% Unknown 14.5% African American 2.6% Other 2.1% Asian or Pacific Islander 1.6% American Indian 1.0%	Mean: 10.6 SD: ±4.2 Range: 2–19	Female: 39.9% Male: 60.1%	Cohort	Ambulatory care developmental paediatrics clinic or child and adolescent psychiatry clinic	≥1 psychotropic medication	NDD 70.48% ANX disorders 19.6% DEP disorders 15.6%
Patel et al. [35] (2024) ^2^	4114	United States	Race/ethnicity groups were imputed by study authors from genetic ancestry proportions: White 82% Hispanic/Latino 7% Black 6% Complex 2% Asian 2%	Median: 30 Range: 0.5–98	Female: 63% Male: 35% Not stated: 2%	Cohort	-	-	MDD 44 GAD 38% ADHD 19% BD 10% PTSD 8% SCZ < 1%
Kanu et al. [31] (2024)	263 on risperidone	United States	White 68.4% Black 24.7% Other 6.8%	Mean: 13.1 Range: 3.0–19.3)	Female: 49.4% Male: 50.6%	Cross-sectional	Hospital-based	Prescribed oral risperidone, oral paliperidone, or intramuscular paliperidone	DEP 30.8% DMDD 26.6% BD 19.8% SCZ 5.3%
	124 on paliperidone		White 54.0% Black 37.1% Other 8.9%	Mean: 14.9 Range: 5.1–18.8)	Female: 44.4% Male: 55.6%				DMDD 26.6% DEP 21.0% BD 20.2% SCZ 9.7%
Holck et al. [28] (2024) ^1^	147	Sweden	-	Mean: 39.1 SD: ±14.5 Range: 18–76	Female: 65.9% Male: 34.1%	Cross-sectional	Several psychiatric clinics	-	SCZ 65.1% ANX 59.5% Dysthymia/Chronic DEP 49.6% PS 41.0% BD 8.4%
De Brabander et al. [22] (2024)	412	Netherlands and Flemish Belgium	White 82.8% Mixed 8.6% Moroccan 3.2% Turkish 2.9% Other 2.5%	Mean: 27.2 SD: ±7.1	Female: 20.4% Male: 79.6%	Cross-sectional	Local psychosis departments or academic centres (both in- and out-patients)	≥1 drug for psychosis	SCZ + related 86.9% Psychosis 10.0% DD 2.4% BD 0.5% Unknown 0.2%
Gerlach et al. [25] (2024)	214	Canada	European 72.4% Mixed 17.3% American 3.7% Asian 2.8% African 1.4% Near Eastern 1.4%	Mean: 12 SD: ±3.8	Female: 36.9% Male: 63.1%	Cross-sectional	Clinical care context	Initiating or changing psychiatric medications + history of amphetamine treatment	-
De Brabander et al. [21] (2025)	708	Netherlands and Flemish Belgium	White 80.9% Mixed 8.8% Not reported 3.1% Turkish 2.8% Moroccan 2.3% Other 2.1%	Mean: 27.8 SD: ±8.2	Female: 24.2% Male: 75.8%	Cohort	Local psychosis departments or academic centres (both in- and out-patients)	-	SCZ + related 80.4% Psychosis 14.7% DD 2.4% BD 1.8% Unknown 0.7%
Gerlach et al. [24] (2025)	1281	Canada	European 69.3% Mixed 14.2% Asian 6.6% American 3.8% African 1.8% Near Eastern 1.7% Unknown 1.3% Latino 1.2% Oceanian 0.1%	Mean: 13.5 SD: ±3.9	Female: 49.3% Male: 50.7%	Cross-sectional	Clinical care context	Initiating or changing psychiatric medications	-
Gras et al. [27](2025)	466	Switzerland	Caucasian 72.7% Mixed 22.1% African 4.7% Asian 0.4%	Median: 37 IQR: 49–28	Female: 56.2% Male: 43.8%	Cohort	Public hospital and a private ambulatory clinic	Treated with oral aripiprazole for ≥2 weeks and/or with long-acting formulations for ≥4 months	Other 33.5% SCZ 33.3% SAD 11.2% BD 9.7% DEP 9.9% Missing 2.6%

BUP, bupropion; SD, standard deviation; SCZ schizophrenia; DD, delusional disorder; BD, bipolar disorder; DEP, depression; PD, personality disorder; NDD, neurodevelopmental disorders; PTSD, post-traumatic stress disorder; ANX, anxiety; ED, eating disorder; SSD, somatic symptom disorder; SAD, schizoaffective disorder; PS, personality syndrome; MDD, major depressive disorder; DMDD, disruptive mood dysregulation disorder; GAD, generalized anxiety disorder; ADHD, attention deficit hyperactivity disorder. ^1^ Characteristics for Holck et al. were taken from the ‘Subjects receiving CYP2D6 substrate/s’ (n = 164) cohort a; there was no specific characteristics for the 147 participants who had genotyping results. ^2^ Characteristics for Patel et al. were only available for the full 15,000-participant cohort; however, only 4114 reported medications and were used to evaluate CYP2D6 phenoconversion. ^3^ Race, ethnicity and genetic ancestry are reported according to the terminology and classifications used in the original studies. Where these results were imputed from genetic ancestry, this is explicitly indicated.

**Table 3:** Binary logistic regression of modified PHQ-4 scores by CYP2D6 phenoconversion status. The results are reported as odds ratios (OR) with 95% confidence intervals. The binary outcome was defined as modified PHQ-4 scores ≥ 6. Reference categories: CYP2D6 normal metaboliser (genotypic phenotype); female (sex); White British or Irish (ethnicity); never smoked (smoking); once or twice a week (alcohol). Model adjusted for genotypic CYP2D6 phenotype, age, sex, ethnicity, Townsend deprivation index, number of self-reported medications, number of self-reported indications, smoking status and alcohol use.

Term	OR	95% CI	*p*-Value
(Intercept)	1.81	1.48–2.22	1.07 × 10^−8^
Phenoconverted	1.19	1.11–1.28	1.85 × 10^−6^
CYP2D6 intermediate metaboliser	1.05	0.99–1.11	0.13
CYP2D6 poor metaboliser	1	0.89–1.13	0.934
CYP2D6 ultrarapid metaboliser	0.97	0.78–1.2	0.788
Age	0.95	0.95–0.96	6.22 × 10^−137^
Male	1.16	1.09–1.23	8.06 × 10^−7^
Ethnicity other	1.27	1.14–1.4	8.37 × 10^−6^
Townsend deprivation	1.06	1.06–1.07	2.16 × 10^−49^
Number of self-reported medications	1.1	1.08–1.11	1.09 × 10^−70^
Number of self-reported indications	1.05	1.04–1.07	1.99 × 10^−13^
Smoking current	1.47	1.36–1.58	4.22 × 10^−24^
Smoking previous	0.91	0.86–0.97	0.005
Alcohol daily or almost daily	0.86	0.78–0.94	6.88 × 10^−4^
Alcohol never	1.27	1.16–1.39	3.81 × 10^−7^
Alcohol one to three times a month	1.09	0.99–1.2	0.086
Alcohol special occasions only	1.3	1.19–1.42	1.18 × 10^−8^
Alcohol three or four times a week	0.81	0.74–0.88	3.54 × 10^−6^

OR, odds ratio; CI, confidence interval.

## Data Availability

Restrictions apply to the availability of these data. Data were obtained from UK Biobank (application number: 48433) and are available from the UK Biobank (https://www.ukbiobank.ac.uk/) upon application and approval. Data used in the meta-analysis were extracted from previously published articles and are available in the public domain. Full details of included studies are provided within the article and its Supplementary Material.

## Supplementary Materials

The following supporting information can be downloaded at https://www.mdpi.com/article/10.3390/pharmaceutics18101319/s1. Figure S1: PRISMA flow diagram; Figure S2: Meta-analysis of CYP2D6 phenoconversion prevalence among individuals with a psychiatric disorder using the logit transformation; Figure S3: Meta-analysis of CYP2D6 phenoconversion prevalence among individuals with a psychiatric disorder using the Freeman–Tukey double arcsine transformation; Figure S4: Doi plot of CYP2D6 phenoconversion prevalence among individuals with a psychiatric disorder using the logit transformation; Figure S5: Galbraith plot of CYP2D6 phenoconversion prevalence among individuals with a psychiatric disorder using the logit transformation; Figure S6: Subgroup analysis of CYP2D6 phenoconversion prevalence among individuals with a psychiatric disorder using the logit transformation by age; Figure S7: Meta-analysis of CYP2D6 phenoconversion prevalence among adults with a psychiatric disorder using the Freeman–Tukey double arcsine transformation; Figure S8: Doi plot of CYP2D6 phenoconversion prevalence among adults with a psychiatric disorder using the logit transformation; Figure S9: Galbraith plot of CYP2D6 phenoconversion prevalence among adults with a psychiatric disorder using the logit transformation; Figure S10: Doi plot of CYP2D6 phenoconversion prevalence among individuals with a psychiatric disorder following removal of Patel et al.; Table S1: PRISMA checklist; Table S2: MOOSE checklist; Table S3: MEDLINE and Embase search strategy; Table S4: PsycINFO search strategy; Table S5: JBI checklist for prevalence studies questions; Table S6: Self-reported psychological and psychiatric problems of UK Biobank cohort; Table S7: CYP2D6 inhibitor use within the UK Biobank cohort; Table S8: Demographics table for the UK Biobank cohort stratified by phenoconversion status; Table S9: Excluded studies and reasons for exclusion; Table S10: Funding sources, conflicts of interest and PMIDs for included studies; Table S11: Study strategies; Table S12: Extracted prevalences of CYP2D6 phenoconversion reported among individuals with a psychiatric disorder; Table S13: JBI checklist for prevalence studies results; Table S14: Leave-one-out analysis of CYP2D6 phenoconversion prevalence among individuals with a psychiatric disorder using the logit transformation; Table S15: Comparison of included and excluded participants by key characteristics; Table S16: Characteristics of the modified PHQ-4 score analysis sample stratified by phenoconversion status; Table S17: Negative binomial regression of modified PHQ-4 total score by CYP2D6 phenoconversion status; Table S18: CYP2D6 substrate–inhibitor interaction table. Supplementary Methods: Supplementary Methods S1: Self-reported psychological/psychiatric problems within the UK Biobank cohort; Supplementary Methods S2: Strong and moderate CYP2D6 inhibitors; Supplementary Methods S3: Review protocol; Supplementary Methods S4: PHQ-4 questions and UK Biobank fields 2050, 2060, 2070 and 2080. Supplementary Results: Supplementary Results S1: Narrative synthesis results. References [9,10,14,16,17,21,22,23,24,25,26,27,28,29,30,31,32,33,34,35,36,37,38,39,40,41,42,43,44,45,46,47,48,49,57,60,61,67,68,69,70,71,72,73,74,75,76,77,78,79,80,81,82,83,84,85,86,87,88,89] are cited in the supplementary materials.

## Abbreviations

The following abbreviations are used in this manuscript:

UK	United Kingdom
PGx	Pharmacogenomics
CPIC	Clinical Pharmacogenetics Implementation Consortium
DPWG	Dutch Pharmacogenetics Working Group
DDGI	Drug–drug–gene Interaction
CYP450	Cytochrome P450
PHQ-4	4-item Patient Health Questionnaire
ICD-10	International Classification of Diseases, 10th Revision
UKB-RAP	UK Biobank Research Analysis Platform
JBI	Joanna Briggs Institute
REML	Restricted Maximum Likelihood
CI	Confidence interval
PI	Prediction interval
LFK index	Luis Furuya-Kanamori index
PHQ-2	2-item Patient Health Questionnaire
GAD-2	2-item Generalized Anxiety Disorder
IRR	Incidence rate ratio
OR	Odds ratio
SD	Standard deviation
IM	Intermediate metaboliser
NM	Normal metaboliser
PM	Poor metaboliser
UM	Ultrarapid metaboliser
