# Supplementary material for: Drug-Induced CYP2D6 Phenoconversion Among Individuals with a Psychiatric Disorder: Prevalence and Association with Psychological Distress
Source: Pharmaceutics. 2026 Oct 17;18(10):1319. doi: 10.3390/pharmaceutics18101319 (PMC13708733; doi:10.3390/pharmaceutics18101319)
Supplement: Supplementary file 1 [file pharmaceutics-18-01319-s001.zip › pharmaceutics-4554662-supplementary.pdf]

Supplementary Materials: Drug-induced CYP2D6 Phenoconversion Among Individuals with a Psychiatric Disorder: Prevalence and Association with Psychological Distress

Ellen R. Mason [1], Mohamed Y. Ali [1], Richéal M. Burns [2,3], Priyank Shukla [1], Aaron Courtenay [4], David S. Gibson [1], Elaine K. Murray [1] and Catriona Kelly [1,*]

[1] Personalised Medicine Centre, School of Medicine, Ulster University, C-TRIC Builiding, Altnagelvin
Hospital Campus, Glenshane Road, Derry/Londonderry BT47 6SB, UK; mason-e2@ulster.ac.uk (E.R.M.);
ali-m36@ulster.ac.uk (M.Y.A.); p.shukla@ulster.ac.uk (P.S.); d.gibson@ulster.ac.uk (D.S.G.);
e.murray@ulster.ac.uk (E.K.M.)

[2] Department of Social Sciences, Faculty of Science, Atlantic Technological University, F91 YW50 Sligo,
Ireland; richeal.burns@atu.ie

[3] Health and Biomedical Research Centre (HEAL), Faculty of Science, Atlantic Technological University,
F91 YW50 Sligo, Ireland

[4] School of Pharmacy and Pharmaceutical Sciences, Ulster University, Coleraine BT52 1SA, UK;
a.courtenay@ulster.ac.uk

* Correspondence: c.kelly@ulster.ac.uk

Contents

Supplementary Methods **4**

Supplementary Methods S1: Self-reported psychological/psychiatric problems within UK Bioank cohort 4

Supplementary Methods S2: Strong and moderate CYP2D6 inhibitors 5

Supplementary Methods S3: Review protocol 6

Supplementary Methods S4: PHQ-4 questions and UK Biobank Fields 2050, 2060, 2070 and 2080 12

Supplementary Results **13**

Supplementary Results S1: Narrative synthesis results 13

Supplementary Tables **15**

Table S1: PRISMA checklist 15

Table S2: MOOSE checklist 18

Table S3: MEDLINE and Embase Search Strategy 20

Table S4: PsycINFO search strategy 20

Table S5: JBI checklist for prevalence studies questions..21
Table S6: Self-reported psychological and psychiatric problems of UK Biobank cohort..22
Table S7: CYP2D6 inhibitor use within the UK Biobank cohort..22
Table S8: Demographics table for UK biobank cohort stratified by phenoconverion status..23
Table S9: Excluded studies and reasons for exclusion..25
Table S10: Funding sources, conflicts of interest and PMIDs for included studies.26
Table S11: Study strategies..27
Table S12: Extracted prevalences of CYP2D6 phenoconversion reported among individuals with a psychiatric disorder..30
Table S13: JBI checklist for prevalence studies results..33
Table S14: Leave-one-out analysis of CYP2D6 phenoconversion prevalence among individuals with a psychiatric disorder using the logit transformation..34
Table S15: Comparison of included and excluded participants by key characteristics..35
Table S16: Characteristics of the modified PHQ-4 score analysis sample stratified by phenoconversion status..36
Table S17: Negative binomial regression of modified PHQ-4 total score by CYP2D6 phenoconversion status..37
Table S18: CYP2D6 substrate-inhibitor interaction table..38
Supplementary Figures..45
Figure S1: PRISMA flow diagram..45
Figure S2: Meta-analysis of CYP2D6 phenoconversion prevalence among individuals with a psychiatric disorder using the logit transformation..46
Figure S3: Meta-analysis of CYP2D6 phenoconversion prevalence among individuals with a psychiatric disorder using the Freeman-Tukey double arcsine transformation..47
Figure S4: Doi plot of CYP2D6 phenoconversion prevalence among individuals with a psychiatric disorder using the logit transformation..48
Figure S5: Galbraith plot of CYP2D6 phenoconversion prevalence among individuals with a psychiatric disorder using the logit transformation..48

Figure S6: Subgroup analysis of CYP2D6 phenoconversion prevalence among individuals with a psychiatric disorder using the logit transformation by age 49
Figure S7: Meta-analysis of CYP2D6 phenoconversion prevalence among adults with a psychiatric disorder using the Freeman-Tukey double arcsine transformation 50
Figure S8: Doi plot of CYP2D6 phenoconversion prevalence among adults with a psychiatric disorder using the logit transformation 51
Figure S9: Galbraith plot of CYP2D6 phenoconversion prevalence among adults with a psychiatric disorder using the logit transformation 51
Figure S10: Doi plot of CYP2D6 phenoconversion prevalence among individuals with a psychiatric disorder following removal of Patel et al. 52
References......53

Supplementary Methods

<u>Supplementary Methods S1: Self-reported psychological/psychiatric problems within UK Bioank cohort</u>

List of self-reported psychological/psychiatric problems recorded at Instance 0 which were included in our analysis.

Psychological/psychiatric problem

- Depression
  - Post-natal depression
- Anxiety/panic attacks
- Nervous breakdown
- Schizophrenia
- Deliberate self-harm/suicide attempt
- Mania/bipolar disorder/manic depression
- Substance abuse/dependency
  - Alcohol dependency

        - Opioid dependency
        - Other substance abuse/dependency
    - Post-traumatic stress disorder
    - Anorexia/bulimia/other eating disorder
    - Stress
    - Obsessive compulsive disorder
    - Insomnia

Supplementary Methods S2: Strong and moderate CYP2D6 inhibitors

These CYP2D6 inhibitors were identified using the 'Drug Interactions Flockhart Table'.[1]

Strong CYP2D6 Inhibitors

- **Bupropion**
    - Brand names: Aplenzin, Auvelity, Budeprion, Contrave, Forfivo, Mysimba, Wellbutrin, Zyban
- **Fluoxetine**
    - Brand names: Prozac, Sarafem, Symbyax, Rapiflux, Selfemra
- **Paroxetine**
    - Brand names: Brisdelle, Paxil, Pexeva
- **Perhexiline**
    - Brand names: —
- **Quinidine**
    - Brand names: Nuedexta, Quin-G, Cardioquin, Quinidex, Quinaglute, Quinora, Quin-Release

Moderate CYP2D6 Inhibitors

- **Abiraterone**
    - Brand names: Abiraterone Accord, Abiraterone Krka, Abiraterone Mylan, Abirtega, Akeega, Yonsa, Zytiga
- **Cinacalcet**
    - Brand names: Cinacalcet Accordpharma, Mimpara, Sensipar
- **Doxepin**
    - Brand names: Prudoxin, Silenor, Sinequan, Zonalon
- **Dronedarone**
    - Brand names: Multaq
- **Duloxetine**
    - Brand names: Cymbalta, Drizalma, Irenka, Yentreve
- **Halofantrine**
    - Brand names: Halfan

- **Mirabegron**
    - Brand names: Myrbetriq
- **Moclobemide**
    - Brand names: Manerix
- **Rolapitant**
    - Brand names: Varubi

List of strong and moderate CYP2D6 inhibitors used in our analysis identified from the Flockhart table and any brand names that were also searched. Brand names were taken from Drugs.com and/or DrugBank.[1]

Supplementary Methods S3: Review protocol

**Review information**	
**Title of the review**	Prevalence of CYP2D6 Phenoconversion Among Individuals with a Psychiatric Disorder: A Systematic Review, Meta-analysis and UK Biobank Validation
**Question**	What is the pooled prevalence of CYP2D6 phenoconversion among individuals with a psychiatric disorder?
**First reviewer**	Ellen Mason
**Team of reviewers**	Mohamed Ali
**Supervisor/Project PI**	Dr Catriona Kelly
**Other Project Supervisors**	Prof David Gibson<br>Dr Elaine Murray<br>Dr Richéal Burns
**Project Title**	Pharmacogenomics of psychiatric medications

Support – please state if advice/training or personnel required at each stage	
**SR overview**	• Atlantic Technological University (ATU) Systematic literature review and meta-analysis (iNOTE) course<br>• Cochrane Interactive Learning: Conducting an Intervention Review
**Protocol development**	• Cochrane Interactive Learning: Conducting an Intervention Review
**Literature searching**	• ATU Systematic literature review and meta-analysis (iNOTE) course<br>• Cochrane Interactive Learning: Conducting an Intervention Review<br>• Appointment with university subject librarian
**Quality appraisal**	• Atlantic Technological University (ATU) Systematic literature review and meta-analysis (iNOTE) course<br>• Cochrane Interactive Learning: Conducting an Intervention Review
**Data extraction**	
**Synthesis**	
**Writing up**	

1. Background to review

Introduction

Pharmacogenomics (PGx) integrates the subjects of pharmacology and genomics to understand how genetic variation can influence the rate of drug metabolism, drug efficacy and the occurrence of adverse drug reactions (ADRs) [2]. The rate of drug metabolism can be altered when genetic variations occur in genes that encode proteins responsible for drug metabolism, such as the cytochrome p450 (CYP450) enzymes [3]. Understanding this genetic variation has led to the classification of individuals into various metaboliser phenotypes including poor, intermediate, normal and ultra-rapid metabolisers through PGx testing. These phenotypes can then be used to stratify medication selection and dosing. However, these metaboliser phenotypes can also be influenced by non-genetic factors such as concomitant medications, smoking and pregnancy [4]. Exposure to these non-genetic factors can induce or inhibit these drug-metabolising enzymes, altering their metabolic activity. This can result in a change to the genotype-predicted metaboliser phenotype, a process known as phenoconversion. Currently, PGx testing does not consider these non-genetic factors when determining metaboliser phenotypes [5]. Not considering these non-genetic factors could potentially result in continued reduced drug efficacy and increased risk of ADRs for individuals exposed to these factors, despite having undergone PGx testing.

CYP2D6 and CYPC19, two highly polymorphic CYP450 enzymes, are two of the most studied genes in PGx due to their substantial involvement in the metabolism of many commonly prescribed medications [6,7]. CYP2D6, in particular, is involved in the drug metabolism of ~25% of commonly used drugs such as anti-depressants and opioids [8]. The CYP2D6 enzyme can be inhibited by various drugs, particularly psychiatric medications. These inhibitors have been categorised based on their ability to raise the plasma concentration of concomitant CYP2D6 substrates by sources such as the Food and Drug Administration (FDA) and the Flockhart table [1,9]. Strong CYP2D6 inhibitors (e.g. bupropion, fluoxetine and paroxetine) can cause a ≥5-fold increase in plasma concentrations; moderate inhibitors (e.g. doxepin, duloxetine and moclobemide) a 2-5-fold increase; and weak inhibitors (e.g. sertraline and escitalopram) an increase of ≤2-fold. These changes in plasma concentrations of CYP2D6 substrates can cause phenoconversion, particularly with strong or moderate inhibitors [10]. Patients with a psychiatric condition could therefore be at increased risk of

pheneoconversion due to the frequent use of these CYP2D6 inhibitors in psychiatric treatment. It is therefore vital that non-genetic factors, such as concomitant medications, are taken into consideration when determining an individual's metaboliser phenotype.

Rationale

Estimating the pooled prevalence of CYP2D6 phenoconversion among individuals with a psychiatric disorder would be beneficial to inform future research on the feasibility of integrating pharmacogenomics-guided prescribing into standard care within psychiatry. For example, its inclusion in future economic models could better reflect real-world circumstances and therefore provide a more comprehensive and accurate economic evaluation.

Aim

To estimate and validate the pooled prevalence of CYP2D6 phenoconversion among individuals with a psychiatric disorder through a meta-analytic synthesis and biobank-based validation.

2. Specific objectives

1. To conduct a meta-analysis to obtain the pooled prevalence of CYP2D6 phenoconversion across eligible studies focusing on individuals with a psychiatric disorder.

2. To validate the prevalence of CYP2D6 phenoconversion in a cohort of individuals with any psychiatric disorder from UK Biobank.

3. a) Criteria for including studies in the review

	Inclusion	Exclusion
**Population**	Individuals diagnosed with any psychiatric disorder	Individuals not diagnosed with a psychiatric disorder
**Outcome**	Prevalence of CYP2D6 phenoconversion either as a single overall prevalence or broken down into changes at a metaboliser phenotype level	Studies not reporting the overall prevalence of CYP2D6 phenoconversion or the changes in metaboliser phenotype breakdown
**Setting**	Clinical, research or population-based studies	Non-human studies or study settings unrelated to psychiatry
**Study design**	Any observational studies (e.g. cross-sectional and cohort studies)	Case reports, reviews, opinions and conference abstracts.

4. Search methods

**Electronic databases**	All studies will be identified through searches conducted in Ovid, using the following electronic databases:<br><br>MEDLINE<br>EMBASE<br>PsycINFO
**Other methods used for identifying relevant research**	Reference checking

5. **Search strategy** - Embase	
**Search #**	**Searches**
1	"phenoconversion" OR "phenoconvert*" OR "phenotype conversion" OR "genotype-phenotype discordance" OR "genotype-phenotype mismatch"
2	mental* OR psychiatr* OR depressi* OR schizo* OR psycho* OR bipolar* OR anxi* OR obsess* OR post-traumat* OR dissociative* OR mood OR affective OR personality or antidepress* or antipsychotic* or anti-depress* or anti-psychotic
3	exp mental health/ or exp mental disease/
4	2 or 3
5	1 and 4

6. **Methods of review**	
**Software to be used**	Covidence will be used for study screening<br>Data extraction form will be in Microsoft Excel<br>Zotero/Covidence will be used to keep track of references<br>Statistical analyses will be performed in Microsoft Excel/R/SPSS
**Study screening**	Studies will be screened by two independent reviewers, Ellen Mason and Mohamed Ali.

	Any discrepancies will be resolved by a consensus discussion or through discussion with a third author.
**Quality assessment**	Quality assessment will be completed using the Joanna Briggs Institute (JBI) checklist for prevalence studies [11]. This tool includes 9 questions which are used to assess methodological quality of prevalence studies.<br><br>If our search produces studies that are not prevalence studies, but report it is a secondary outcome, we will still use the JBI prevalence critical appraisal tool to assess the quality of the prevalence data required for our review with adaptions to the questions where required.
**Data extraction**	Data extraction will be carried out by one reviewer, Ellen Mason, and checked by a second independent reviewer, Mohamed Ali.<br><br>Data to be extracted from eligible studies will include:<br>• **Study and patient characteristics**<br>o PMID<br>o First author<br>o Year of publication<br>o Sample size<br>o Country<br>o Ethnicity<br>o Age group<br>o Sex distribution<br>o Cohort source<br>o Main diagnoses<br>o Drug(s) of interest<br>o Study design<br>o Funding source<br>o Conflicts of interest<br><br>• **Genotype, phenotype and phenoconversion data**<br>o Method of genotyping<br>o CYP2D6 alleles tested<br>o Source of CYP2D6 inhibitors used e.g. FDA, Flockhart table<br>o How was phenoconversion determined e.g. algorithm used or definition<br>o Genotype-based phenotype distribution<br>o Post phenoconversion phenotype distribution<br>o Overall phenoconversion prevalence
**Narrative synthesis**	In our narrative synthesis we will summarise and interpret the findings from the data extraction tables. This will allow us to provide context to the potential distribution of phenoconversion prevalence results and explore different methodologies and possible sources of heterogeneity.

**Meta-analysis**	To conduct our meta-analysis:<br>• The prevalence of CYP2D6 phenoconversion and sample size will be extracted from all eligible studies and converted to a value between 0 and 1, if not already provided.<br>• We will then apply the logit transformation to stabilise the variance using the equations provided in the methodology by Barendregt and colleagues, using MetaXL software on Microsoft Excel [12].<br>• However, if the prevalences extracted are too close to 0 or 1, we will instead use the Freeman–Tukey double-arcsine square root transformation, which handles these extreme prevalences better.
**Publication bias**	Publication bias will be assessed using:<br>• Egger's regression test<br>• Funnel plot<br>• LFK index and Doi plots
**Heterogeneity**	Heterogeneity will be assessed using:<br>• I^2 statistic<br>• Cochran's Q test<br>• Galbraith plot<br>• Prediction intervals<br><br>Subgroup analyses, sensitivity analyses and meta-regression will be performed to further explore heterogeneity if the number of studies included in the review allows.<br><br>A random-effects model will be used if there is substantial heterogeneity present, otherwise a fixed-effects model will be used.
**Grading evidence**	GRADE assessment of certainty is the primary method of grading evidence in systematic reviews, however it was designed for evaluating intervention effects [13].<br><br>The primary outcome of this review is prevalence and therefore formal GRADE assessment is not directly applicable. Instead, we will discuss the domains of GRADE narratively and supplemented with any other relevant analysis e.g. Eggers test and funnel plot for publication bias. The 5 GRADE domains are:<br>• Risk of bias<br>• Inconsistency<br>• Indirectness<br>• Imprecision<br>• Publication bias
**Validation**	Our meta-analysis results will be validated in a cohort of individuals with any psychiatric condition from UK Biobank. To do this we will:

	• Create a subset of participants who have any psychiatric condition<br>• Determine each participant's CYP2D6 metaboliser phenotype<br>• Identify participants prescribed a strong/moderate CYP2D6 inhibitor<br>• Use the phenoconversion application algorithm, as described in the tutorial by Cicali and colleagues, to identify participants who will have likely undergone phenoconversion based on their prescribed medications [14].<br>• Calculate the overall estimated prevalence of phenoconversion within the cohort

**7. Presentation of results**	
**Additional material**	Protocol<br>PRISMA Flow chart<br>Data extraction tables<br>Forest plots
**Outputs from review**	Research article<br>Poster<br>Presentation

**8. Timeline for review – when do you aim to complete each stage of the review**	
**Protocol**	1-2 weeks
**Literature searching**	1-2 weeks
**Quality appraisal**	2 weeks
**Data extraction**	2 weeks
**Synthesis**	2 weeks
**Writing up**	2 weeks

Supplementary Methods S4: PHQ-4 questions and UK Biobank Fields 2050, 2060, 2070 and 2080

PHQ-4 questions

**Over the last two weeks, how often have you been bothered by the following problems?**	**Not at all**	**Several days**	**More than half the days**	**Nearly every day**	**Subscale category**
Feeling nervous, anxious or on edge	0	1	2	3	Anxiety (GAD-2)
Not being able to stop or control worrying	0	1	2	3	
Little interest or pleasure in doing things	0	1	2	3	Depression (PHQ-2)
Feeling down, depressed, or hopeless	0	1	2	3	
**Total**					

PHQ-4 Scoring: Total score ranges from 0 to 12, with categories of psychological distress as follows:

- None: 0-2
- Mild: 3-5
- Moderate: 6-8
- Severe: 9-12

UK Biobank Fields 2050, 2060, 2070 and 2080

UK Biobank Field	Over the past two weeks, how often have you…	Not at all	Several days	More than half the days	Nearly every day	Do not know	Prefer not to answer
2050	Felt down, depressed or hopeless?						
2060	Had little interest or pleasure in doing things?						
2070	Felt tense, fidgety or restless?						
2080	Felt tired or had little energy?						

Supplementary Results

Supplementary Results S1: Narrative synthesis results

There was a predominance of recent research (*n* = 16, 89%) into CYP2D6 phenoconversion among individuals with a psychiatric disorder within the past five years (Table 1). The included studies were carried out in Europe (*n* = 9, 50%), the United States (US) (*n* = 5, 28%), Canada (*n* = 2, 11%) and Australia (*n* = 2, 11%), all with a predominantly white European/Caucasian population. Sample sizes ranged from 60 to 4 114 participants. Based on published prevalence estimates of 10-30%, a minimum sample size of 139 participants was calculated (95% confidence and 5% precision) using the Scalex SP calculator.[15,16] Most studies (*n* = 12, 67%) met this threshold. The majority of studies were also carried out in an adult population (≥19 years old) (*n* = 12, 67%), with five studies looking at CYP2D6 phenoconversion in paediatric/adolescent (<19 years old) populations (*n* = 5, 28%), and one study looking at a population ranging from 0.5 to 98 years old.[16] All included studies were observational, adopting either a cross-sectional (*n* = 12, 67%) or cohort (*n* = 6, 33%) design. Funding sources and conflicts of interest for included studies are reported in supplementary table 9.

Seventeen studies (94%) used valid genotyping methods however one study provided insufficient detail to determine validity (supplementary table 10).[17] Of the 15 studies reporting tested alleles, all genotyped for CYP2D6 single nucleotide polymorphisms (SNPs) and the *5 deletion, eleven (73%) for copy number variations (CNVs) and three (20%) for hybrid alleles. The predominant genotype-to-phenotype translation method used was the standardised CPIC/DPWG guidelines (*n* = 11, 61%), published in 2019.[18] CYP2D6

phenoconversion was determined using the Borges et al., standard multiplication factor in most studies (*n* = 12, 67%). The remaining studies used a metabolic-ratio approach (*n* = 2, 11%)[19,20], stepwise phenotype reduction based on inhibitor strength (*n* = 2, 11%)[17,21], conversion of all participants on a strong inhibitor to poor metabolisers (*n* = 1, 6%)[22] or a combination of multiple methods (*n* = 1, 6%).[23] Most studies (*n* = 14, 78%) considered both strong and moderate CYP2D6 inhibitors, with the Flockhart table being the most frequently used source (*n* = 10, 56%), either exclusively, or alongside other sources.

All 18 studies were appraised using the JBI Critical Appraisal Checklist for Studies Reporting Prevalence Data (supplementary table 11).[24] Across the seven applicable items, the overall methodological quality was high. All studies appropriately defined their sample frame, recruited patients in an appropriate way to estimate CYP2D6 phenoconversion in a psychiatric cohort, described the subjects and setting in sufficient detail and used valid methods to define phenoconversion. Sample size (item 3) was not considered to be adequate in six studies, one study did not carry out genotyping in a standard way throughout the study period (item 7) and one study only carried out descriptive statistics so was considered not to have appropriate statistical analysis (item 8). No studies were excluded on the basis of quality assessment.

The prevalence of CYP2D6 phenoconversion ranged from 6% to 50% across studies, with considerable variation likely reflecting differences in study populations and methods (Table 2). Eleven (61%) studies detailed how many participants were on at least one CYP2D6 inhibitor with this ranging from 10% to 78%. The most frequently prescribed CYP2D6 inhibitors across all studies included the strong inhibitors bupropion, fluoxetine and paroxetine. Eleven (61%) studies directly reported the number of participants who phenoconverted.[16,17,19,21,23,25–30] The remaining seven (39%) studies provided the metaboliser phenotype distribution before and after CYP2D6 phenoconversion, allowing a net change to be calculated to estimate the prevalence.[20,22,31–35]

Supplementary Tables

Table S1: PRISMA checklist

Section and Topic	Item #	Checklist item	Location where item is reported
**TITLE**			
Title	1	Identify the report as a systematic review.	Not in title as used as validation
**ABSTRACT**			
Abstract	2	See the PRISMA 2020 for Abstracts checklist.	Abstract follows 'original article' structure
**INTRODUCTION**			
Rationale	3	Describe the rationale for the review in the context of existing knowledge.	Page 5
Objectives	4	Provide an explicit statement of the objective(s) or question(s) the review addresses.	Pages 5-6
**METHODS**			
Eligibility criteria	5	Specify the inclusion and exclusion criteria for the review and how studies were grouped for the syntheses.	Supplementary page 11
Information sources	6	Specify all databases, registers, websites, organisations, reference lists and other sources searched or consulted to identify studies. Specify the date when each source was last searched or consulted.	Supplementary page 11
Search strategy	7	Present the full search strategies for all databases, registers and websites, including any filters and limits used.	Supplementary tables 2-3
Selection process	8	Specify the methods used to decide whether a study met the inclusion criteria of the review, including how many reviewers screened each record and each report retrieved, whether they worked independently, and if applicable, details of automation tools used in the process.	Supplementary page 11
Data collection process	9	Specify the methods used to collect data from reports, including how many reviewers collected data from each report, whether they worked independently, any processes for obtaining or confirming data from study investigators, and if applicable, details of automation tools used in the process.	Supplementary page 11
Data items	10a	List and define all outcomes for which data were sought. Specify whether all results that were compatible with each outcome domain in each study were sought (e.g. for all measures, time points, analyses), and if not, the methods used to decide which results to collect.	Supplementary page 11

Section and Topic	Item #	Checklist item	Location where item is reported
	10b	List and define all other variables for which data were sought (e.g. participant and intervention characteristics, funding sources). Describe any assumptions made about any missing or unclear information.	Supplementary page 8
Study risk of bias assessment	11	Specify the methods used to assess risk of bias in the included studies, including details of the tool(s) used, how many reviewers assessed each study and whether they worked independently, and if applicable, details of automation tools used in the process.	Supplementary page 11
Effect measures	12	Specify for each outcome the effect measure(s) (e.g. risk ratio, mean difference) used in the synthesis or presentation of results.	Page 8 and supplementary page 9
Synthesis methods	13a	Describe the processes used to decide which studies were eligible for each synthesis (e.g. tabulating the study intervention characteristics and comparing against the planned groups for each synthesis (item #5)).	Supplementary page 11
	13b	Describe any methods required to prepare the data for presentation or synthesis, such as handling of missing summary statistics, or data conversions.	Page 8 and supplementary page 11
	13c	Describe any methods used to tabulate or visually display results of individual studies and syntheses.	Supplementary page 8
	13d	Describe any methods used to synthesize results and provide a rationale for the choice(s). If meta-analysis was performed, describe the model(s), method(s) to identify the presence and extent of statistical heterogeneity, and software package(s) used.	Pages 8-9
	13e	Describe any methods used to explore possible causes of heterogeneity among study results (e.g. subgroup analysis, meta-regression).	Supplementary page 9
	13f	Describe any sensitivity analyses conducted to assess robustness of the synthesized results.	Supplementary page 9
Reporting bias assessment	14	Describe any methods used to assess risk of bias due to missing results in a synthesis (arising from reporting biases).	Supplementary page 9
Certainty assessment	15	Describe any methods used to assess certainty (or confidence) in the body of evidence for an outcome.	Supplementary page 9 and 11
**RESULTS**			
Study selection	16a	Describe the results of the search and selection process, from the number of records identified in the search to the number of studies included in the review, ideally using a flow diagram.	Supplementary figure 1
	16b	Cite studies that might appear to meet the inclusion criteria, but which were excluded, and explain why they were excluded.	Supplementary table 8

Section and Topic	Item #	Checklist item	Location where item is reported
Study characteristics	17	Cite each included study and present its characteristics.	Supplementary results, tables 1-2 and supplementary table 9
Risk of bias in studies	18	Present assessments of risk of bias for each included study.	Supplementary table 11
Results of individual studies	19	For all outcomes, present, for each study: (a) summary statistics for each group (where appropriate) and (b) an effect estimate and its precision (e.g. confidence/credible interval), ideally using structured tables or plots.	Table 2
Results of syntheses	20a	For each synthesis, briefly summarise the characteristics and risk of bias among contributing studies.	Table 1 and supplementary table 11
	20b	Present results of all statistical syntheses conducted. If meta-analysis was done, present for each the summary estimate and its precision (e.g. confidence/credible interval) and measures of statistical heterogeneity. If comparing groups, describe the direction of the effect.	Page 12, figure 2 and supplementary figure 1
	20c	Present results of all investigations of possible causes of heterogeneity among study results.	Page 12, supplementary figures 5, 6, 9 and supplementary table 12
	20d	Present results of all sensitivity analyses conducted to assess the robustness of the synthesized results.	Supplementary figures 3, 6 and 7
Reporting biases	21	Present assessments of risk of bias due to missing results (arising from reporting biases) for each synthesis assessed.	Supplementary figures 4, 8 and 10
Certainty of evidence	22	Present assessments of certainty (or confidence) in the body of evidence for each outcome assessed.	Pages 12-13
**DISCUSSION**			
Discussion	23a	Provide a general interpretation of the results in the context of other evidence.	Page 14
	23b	Discuss any limitations of the evidence included in the review.	Page 18
	23c	Discuss any limitations of the review processes used.	Page 18

Section and Topic	Item #	Checklist item	Location where item is reported
	23d	Discuss implications of the results for practice, policy, and future research.	Page 18
**OTHER INFORMATION**			
Registration and protocol	24a	Provide registration information for the review, including register name and registration number, or state that the review was not registered.	Page 8
	24b	Indicate where the review protocol can be accessed, or state that a protocol was not prepared.	Page 8
	24c	Describe and explain any amendments to information provided at registration or in the protocol.	N/A
Support	25	Describe sources of financial or non-financial support for the review, and the role of the funders or sponsors in the review.	Page 18
Competing interests	26	Declare any competing interests of review authors.	Page 19
Availability of data, code and other materials	27	Report which of the following are publicly available and where they can be found: template data collection forms; data extracted from included studies; data used for all analyses; analytic code; any other materials used in the review.	N/A

From: Page MJ, McKenzie JE, Bossuyt PM, Boutron I, Hoffmann TC, Mulrow CD, et al. The PRISMA 2020 statement: an updated guideline for reporting systematic reviews. BMJ 2021;372:n71. doi: 10.1136/bmj.n71. This work is licensed under CC BY 4.0. To view a copy of this license, visit https://creativecommons.org/licenses/by/4.0/

Table S2: MOOSE checklist

Reporting Criteria	Reported (Yes/No)	Reported on Page No.
**Reporting of Background**		
Problem definition	Yes	4-5
Hypothesis statement	Yes	5-6
Description of Study Outcome(s)	Yes	5-6
Type of exposure or intervention used	Yes	5-6
Type of study design used	Yes	Supp page 12
Study population	Yes	Supp page 12
**Reporting of Search Strategy**		
Qualifications of searchers (eg, librarians and investigators)	Yes	Supp page 12
Search strategy, including time period included in the synthesis and keywords	Yes	6 and supp page 12
Effort to include all available studies, including contact with authors	Yes	Supp page 12
Databases and registries searched	Yes	Supp page 12
Search software used, name and version, including special features used (eg, explosion)	Yes	Supp page 12
Use of hand searching (eg, reference lists of obtained articles)	Yes	Supp page 12
List of citations located and those excluded, including justification	Yes	11 and supp page 12
Method for addressing articles published in languages other than English	Yes	Supp page 12
Method of handling abstracts and unpublished studies	Yes	Supp page 12
Description of any contact with authors	N/A	N/A
**Reporting of Methods**		
Description of relevance or appropriateness of studies assembled for assessing the hypothesis to be tested	Yes	Supp page 12
Rationale for the selection and coding of data (eg, sound clinical principles or convenience)	Yes	Supp page 12
Documentation of how data were classified and coded (eg, multiple raters, blinding, and interrater reliability)	Yes	Supp page 12

Assessment of confounding (eg, comparability of cases and controls in studies where appropriate	Yes	Supp page 12
**Reporting Criteria**	**Reported (Yes/No)**	**Reported on Page No.**
Assessment of study quality, including blinding of quality assessors; stratification or regression on possible predictors of study results	Yes	Supp page 12
Assessment of heterogeneity	Yes	9
Description of statistical methods (eg, complete description of fixed or random effects models, justification of whether the chosen models account for predictors of study results, dose-response models, or cumulative meta-analysis) in sufficient detail to be replicated	Yes	8-9
Provision of appropriate tables and graphics	Yes	Supp page 12
**Reporting of Results**		
Table giving descriptive information for each study included	Yes	Table 1 and supp tables 10-12
Results of sensitivity testing (eg, subgroup analysis)	Yes	12
Indication of statistical uncertainty of findings	Yes	11-12
**Reporting of Discussion**		
Quantitative assessment of bias (eg, publication bias)	Yes	12-13
Justification for exclusion (eg, exclusion of non–English-language citations)	Yes	Supp page 12
Assessment of quality of included studies	Yes	12-13
**Reporting of Conclusions**		
Consideration of alternative explanations for observed results	Yes	18
Generalization of the conclusions (ie, appropriate for the data presented and within the domain of the literature review)	Yes	17-18
Guidelines for future research	Yes	18
Disclosure of funding source	Yes	19

Table S3: MEDLINE and Embase Search Strategy

**1. Search strategy** – Medline/Embase	
**Search #**	**Searches**
1	"phenoconversion" OR "phenoconvert*" OR "phenotype conversion" OR "genotype-phenotype discordance" OR "genotype-phenotype mismatch"
2	mental* OR psychiatr* OR depressi* OR schizo* OR psycho* OR bipolar* OR anxi* OR obsess* OR post-traumat* OR dissociative* OR mood OR affective OR personality or antidepress* or antipsychotic* or anti-depress* or anti-psychotic
3	exp mental health/ or exp mental disease/
4	2 or 3
5	1 and 4

Table S4: PsycINFO search strategy

**9. Search strategy** – PsycINFO	
**Search #**	**Searches**
1	"phenoconversion" OR "phenoconvert*" OR "phenotype conversion" OR "genotype-phenotype discordance" OR "genotype-phenotype mismatch"
2	mental* OR psychiatr* OR depressi* OR schizo* OR psycho* OR bipolar* OR anxi* OR obsess* OR post-traumat* OR dissociative* OR mood OR affective OR personality or antidepress* or antipsychotic* or anti-depress* or anti-psychotic
3	exp mental health/
4	2 or 3
5	

	1 and 4

Table S5: JBI checklist for prevalence studies questions

	**Question**
1	Was the sample frame appropriate to address the target population?
2	Were study participants recruited in an appropriate way?
3	Was the sample size adequate?
4	Were the study subjects and setting described in detail?
5	Was data analysis conducted with sufficient coverage of the identified sample?
6	Were valid methods used for the identification of the condition?
7	Was the condition measured in a standard, reliable way for all participants?
8	Was there appropriate statistical analysis?
9	Was the response rate adequate, and if not, was the low response rate managed appropriately?

Some of the JBI checklist questions are particularly relevant for prevalence studies. Items 5 and 9 focus on coverage of the sample and response rate, which were not applicable to the studies included in this review as these items are relevant to typical prevalence studies and none of the studies in this review evaluated the prevalence of CYP2D6 phenoconversion as their primary aim. Item 2 was also adapted to assess whether the clinical population was representative of the psychiatric population of interest, rather than requiring sampling methods typical of prevalence studies.

Table S6: Self-reported psychological and psychiatric problems of UK Biobank cohort

Condition	N participants[1]
Depression	26657
Anxiety/panic attacks	6344
Mania/bipolar disorder/manic depression	1342
Alcohol dependency	694
Nervous breakdown	680
Schizophrenia	572
Stress	528
Insomnia	460
Psychological/psychiatric problem	452
Post-natal depression	354
Anorexia/bulimia/other eating disorder	345
Post-traumatic stress disorder	325
Deliberate self-harm/suicide attempt	210
Obsessive compulsive disorder	102
Other substance abuse/dependency	50
Opioid dependency	36

[1] Total number of participants exceed the total size of the cohort (*n*=35 338) due to multi-morbidity.

Table S7: CYP2D6 inhibitor use within the UK Biobank cohort

CYP2D6 Inhibitor	N participants
Fluoxetine	4 895
Paroxetine	1 254
Duloxetine	304
Moclobemide	32
Doxepin	17
Bupropion	14
Quinidine	<5

[1] Total Number of participants exceed the number of participants on a CYP2D6 inhibitor (*n*=6 508) as eleven participants were on two inhibitors.

Table S8: Demographics table for UK biobank cohort stratified by phenoconverion status

	level	Overall	Not phenoconverted	Phenoconverted	p
n		35337	29395	5942	
Age (mean (SD))		55.55 (7.85)	55.60 (7.87)	55.31 (7.75)	0.008
Sex (n (mean)	Female	22710 (64.3)	18569 (63.2)	4141 (69.7)	<0.001
	Male	12627 (35.7)	10826 (36.8)	1801 (30.3)	
Townsend_deprivation (mean (SD))		-0.64 (3.39)	-0.65 (3.40)	-0.64 (3.32)	0.888
Ethnicity (%)	African	103 (0.3)	88 (0.3)	15 (0.3)	0.556
	Any other Asian background	80 (0.2)	67 (0.2)	13 (0.2)	
	Any other Black background	10 (0.0)	8 (0.0)	<5 (0.0)	
	Any other mixed background	92 (0.3)	78 (0.3)	14 (0.2)	
	Any other white background	1119 (3.2)	941 (3.2)	178 (3.0)	
	Asian or Asian British	<5 (0.0)	<5 (0.0)	<5 (0.0)	
	Bangladeshi	8 (0.0)	7 (0.0)	<5 (0.0)	
	Black or Black British	<5 (0.0)	<5 (0.0)	<5 (0.0)	
	British	31640 (89.5)	26266 (89.4)	5374 (90.4)	
	Caribbean	185 (0.5)	157 (0.5)	28 (0.5)	
	Chinese	31 (0.1)	26 (0.1)	5 (0.1)	
	Do not know	25 (0.1)	23 (0.1)	<5 (0.0)	
	Indian	242 (0.7)	212 (0.7)	30 (0.5)	
	Irish	1039 (2.9)	855 (2.9)	184 (3.1)	
	Missing	41 (0.1)	35 (0.1)	6 (0.1)	
	Mixed	11 (0.0)	9 (0.0)	<5 (0.0)	
	Other ethnic group	280 (0.8)	247 (0.8)	33 (0.6)	
	Pakistani	106 (0.3)	94 (0.3)	12 (0.2)	
	Prefer not to answer	124 (0.4)	106 (0.4)	18 (0.3)	
	White	51 (0.1)	42 (0.1)	9 (0.2)	
	White and Asian	62 (0.2)	55 (0.2)	7 (0.1)	
	White and Black African	34 (0.1)	31 (0.1)	<5 (0.1)	

	White and Black Caribbean	49 (0.1)	43 (0.1)	6 (0.1)	
Smoking status (%)	Current	6017 (17.0)	4963 (16.9)	1054 (17.7)	<0.001
	Missing	41 (0.1)	35 (0.1)	6 (0.1)	
	Never	17006 (48.1)	14310 (48.7)	2696 (45.4)	
	Prefer not to answer	137 (0.4)	117 (0.4)	20 (0.3)	
	Previous	12136 (34.3)	9970 (33.9)	2166 (36.5)	
Alcohol intake frequency (%)	Daily or almost daily	6704 (19.0)	5569 (18.9)	1135 (19.1)	0.064
	Missing	41 (0.1)	35 (0.1)	6 (0.1)	
	Never	4755 (13.5)	4010 (13.6)	745 (12.5)	
	Once or twice a week	7811 (22.1)	6497 (22.1)	1314 (22.1)	
	One to three times a month	4191 (11.9)	3423 (11.6)	768 (12.9)	
	Prefer not to answer	88 (0.2)	70 (0.2)	18 (0.3)	
	Special occasions only	5376 (15.2)	4463 (15.2)	913 (15.4)	
	Three or four times a week	6371 (18.0)	5328 (18.1)	1043 (17.6)	
Number of self-reported indications (mean (SD))		3.57 (2.33)	3.55 (2.32)	3.70 (2.39)	<0.001
Number of self-reported medications (mean (SD))		4.99 (3.29)	4.83 (3.24)	5.76 (3.39)	<0.001

SD standard deviation

Baseline characteristics of the UK Biobank cohort with a psychiatric/psychological condition at instance 0 stratified by CYP2D6 phenoconversion status.

Values are represented as a mean (standard deviation) or n (%).

Table S9: Excluded studies and reasons for exclusion

Excluded study	Reason for exclusion
Kiss 2020[36]	Only reported number of participants on CYP2D6 inhibitors
Zourkova 2008[37]	Conference abstract
Mostafa 2019[38]	Cohort was not exclusively individuals with a psychiatric disorder
Kane 2010	DOI could not be found
Ishtiak-Ahmed 2022[39]	Conference abstract
Gill 2022[40]	Cohort was not exclusively individuals with a psychiatric disorder
Berm 2016[41]	This was a Randomised Clinical Trial and we stated in our inclusion criteria that we would only include observational studies
Fan 2022[42]	Publication was not available in English
Shilbayeh 2024[43]	Did not report the number or proportion of individuals who phenoconverted
Eap 2025[44]	Conference abstract
Berm 2015[45]	Conference abstract
Magalhaes 2020[46]	Only reported number of drug interactions that were inhibitory

Table S10: Funding sources, conflicts of interest and PMIDs for included studies

Author (Year)	PMID	Funding source	Conflicts of Interest
Preskorn (2013)	23541126	Private	Yes
Patteet (2015)	26514968	No statement	No statement
Lesche (2020)	31616047	Public and private	Yes
Hole (2021)	33905640	No statement	No statement
Mostafa (2021)	34489765	Public	Yes
Jallaq (2021)	32845723	No funding	Yes
Gloor (2022)	35653884	Private	None declared
den Uil (2023)	37693320	No funding statement	None declared
Scherf-Clavel (2023)	37166747	Public	Yes
Tonti (2024)	38377527	No funding	None declared
Patel (2024)	38783055	No statement	Yes
Kanu (2024)	38377522	No funding	Yes
Holck (2024)	38236047	Public	Yes
De Brabander (2024)	39310932	Public and private	None declared
Gerlach (2024)	38517706	Public and private	Yes
De Brabander (2025)	40228694	Public and private	Yes

Gerlach (2025)	39686785	Private	Yes
Gras (2025)	40662264	Public	None declared

Table S11: Study strategies

Author (Year)	Genotyping method	Alleles tested	SNP	*5	CNV	Hybrid	Method to determine genotype-predicted phenotype	Phenoconversion determination	Inhibitor strength considered	Source of inhibitors
Preskorn (2013)	Pyrosequencing + TaqMan assays	*1, *3, *4, *5, *6, *7, *8, *10, *17, *29, *40, *41, and *xN	X	X	X	-	Star allele-based classification	Metabolic ratio based	-	Literature
Patteet (2015)	Luminex xTAG CYP2D6 Kit v3	*1, *2, *3, *4, *5, *6, *7, *8, *9, *10, *11, *15, *17, *29, *35, *41 and *xN	X	X	X	-	Gaedigk et al system with a further division into EM-s + EM-f subgroups	Borges standard multiplication factor	Strong + moderate	-
Lesche (2020)	TaqMan-based assays	*1, *2, *3, *4, *5, *6, *7, *8, *9, *10, *14A & B, *17, *36, *41, and *xN	X	X	X	*36	Gaedigk et al system	Borges standard multiplication factor	Strong + moderate	Flockhart Table
Hole (2021)	TaqMan qPCR assay	*3, *4, *5, *6, *9, *10, *41 and *xN	X	X	X	-	Star allele-based classification	Metabolic ratio based	Only bupropion	-
Mostafa (2021)	Agena MassArray Iplex + qPCR + long-range PCR	*2, *3, *4, *5, *6, *7, *8, *9, *10, *14A, *14B, *17, *20, *39, and *41	X	X	-	-	CPIC/DPWG AS system	Borges standard multiplication factor	Strong + moderate	FDA table, Flockhart Table and a criteria-based classification by Polasek and colleagues
Jallaq (2021)	The TaqMan Low Density Array + long-range PCR	*2A, *3, *4, *5, *6, *7, *8, *9, *10, *11, *14, *15, *17, *18,*19, *20, *40, *41, *42, and *44	X	X	-	-	CPIC/DPWG AS system	Borges standard multiplication factor	Strong + moderate	-
Gloor (2022)	Custom OpenArray panel + TaqMan CNV qPCR	*1, *2, *3, *4, *5, *6, *7, *8, *9, *10, *17, *29, *35 and *41	X	X	X	-	CPIC/DPWG AS system	Three methods: (1) Activity score modulation according to Lesche et al (2) UF phenoconversion calculator (3) Sequence2Script	Strong + moderate	Geneva University Hospital Drug interactions, cytochromes P450

										and P-glycoprotein list
den Uil (2023)	Methods not specified	-	-	-	-	-	DPWG phenotype translation guidelines	Strong: All to PM except NM → IM<br>Moderate: ↓ level (except PM)	Strong + moderate	Flockhart Table
Scherf-Clavel (2023)	Agena MassArray + RFLP + CYP2D6 CNV Assay	*1, *2A, *2B, *2C, *3, *4, *4J, *4K, *4M, *4N, *4P, *5, *6A, *6C, *9, *10A, *10B, *10C, *14, *17, *34, *35A, *35B, *39, *41, *46A, *46B, *46C, *58, *64, *69, *71, *82, *88 and *114	X	X	X	-	CPIC/DPWG AS system	Borges standard multiplication factor	Strong + moderate	Flockhart Table
Tonti (2024)	OneOme RightMed Comprehensive test	*2, *3, *4, *5, *6, *7, *8, *9, *10, *11, *12, *13, *14, *15, *17, *18, *19, *29, *31, *34, *35, *36, *39, *41, *42, *59, *63, *64, *65, *68, *69, *70, *82, *83, *91, *109, *114, *119, and *xN	X	X	X	X	CPIC/DPWG AS system	Borges standard multiplication factor	Strong + moderate	-
Patel (2024)	Tempus nP (NGS WES + Agena MassARRAY)	*1, *2, *3, *4, *5, *6, *7, *8, *9, *10, *11, *12, *14, *15, *17, *29, *35, *40, *41, and *xN	X	X	X	-	CPIC/DPWG AS system	Borges standard multiplication factor	Strong + moderate	Flockhart Table
Kanu (2024)	TaqMan low density array with long-range PCR/Agena VeriDose assay with CYP2D6 CNV assay	*2, *2A, *3, *4, *5, *6, *9, *14, *15, *17, *19, *29, *40, *41, and *xN	X	X	X	-	CPIC/DPWG AS system	Borges standard multiplication factor	Strong	-
Holck (2024)	Pyrosequencing + TaqMan Drug Metabolism Genotyping	*3, *4, *6, *41 and *xN	X	X	X	-	CPIC/DPWG AS system	Borges standard multiplication factor	Strong + moderate	Flockhart Table
De Brabander (2024)	Custom Illumina SNP array	-	X	-	-	-	Stargazer + PharmGKB validation	Borges standard multiplication factor	Strong + moderate	Flockhart Table
Gerlach (2024)	Agena Veridose custom assay + CYP2D6 Veridose CNV assay	*2, *3, *4, *5, *6, *7, *8, *9, *10, *11, *12, *13, *`4, *15, *17, *18, *19, *20, *22, *29, *35, *36, *40, *41, *44, *68,	X	X	X	*13, *36, *68	CPIC/DPWG AS system	Borges standard multiplication factor	Strong + moderate	Flockhart Table

		*69, *114, *xN and CNV probes								
De Brabander (2025)	Custom Illumina SNP array	-	X	-	-	-	Stargazer + PharmGKB validation	Strong: All to PM except NM → IM Moderate: ↓ level (except PM)	Strong + moderate	Flockhart Table
Gerlach (2025)	Agena Veridose custom assay + CYP2D6 Veridose CNV assay	*2, *3, *4, *5, *6, *7, *8, *9, *10, *11, *12, *13, *`4, *15, *17, *18, *19, *20, *22, *29, *35, *36, *40, *41, *44, *68, *69, *114, *xN and CNV probes	X	X	X	*13, *36, *68	CPIC/DPWG AS system	Borges standard multiplication factor	Strong + moderate	Flockhart Table + Sequence2Script
Gras (2025)	Illumina Global Screening Array + TaqMan CNV qPCR	*3, *4, *5, *6, *10, *41 and *xN	X	X	X	-	CPIC/DPWG AS system	Strong: Converted to PM	Strong	-

SNP Single Nucleotide Polymorphism, *CNV* Copy Number Variation, *qPCR* Quantitative Polymerase Chain Reaction, *NGS* Next Generation Sequencing, *WES* Whole-Exome Sequencing, *RFLP* Restriction Fragment Length Polymorphism, *CPIC* Clinical Pharmacogenetics Implementation Consortium, *DPWG* Dutch Pharmacogenetics Working Group, *AS* Activity Score, *NM* Normal metaboliser, *PM* Poor Metaboliser, *EM-s* Extensive Metaboliser with Slow Activity, *EM-f* Extensive Metaboliser with Fast Activity, *IM* Intermediate Metaboliser, *UM* Ultrarapid Metaboliser, *UF* University of Florida, *ODV:VEN* O-desmethylvenlafaxine: venlafaxine, *ODV:NDV* O-desmethylvenlafaxine:N-desmethylvenlafaxine

Methods used to determine CYP2D6 genotype, genotype-predicted phenotype and assess phenoconversion across studies.

Table S12: Extracted prevalences of CYP2D6 phenoconversion reported among individuals with a psychiatric disorder.

**Author (Year)**	**Sample size**	**Individuals on ≥1 CYP2D6 inhibitor N (%)**	**Inhibitors used N (%)**	**Genotype-predicted phenotype N (%)**	**Phenoconversion-adjusted phenotypes N (%)**	**Individuals who phenoconverted**	**Prevalence (%)**
Preskorn (2013)	900	-	-	-	-	218	24.2%
Patteet (2015)	82	8 (9.76%)	Paroxetine (3, 4%)<br>Bupropion (2, 2%)<br>Sertraline (2, 2%)<br>Duloxetine (1, 1%)	PM: 7 (8.5%)<br>IM: 3 (3.7%)<br>NM: 67 (81.6%)<br>UM: 5 (6.1%)	PM: 12 (14.63%)<br>IM: 5 (6.10%)<br>NM: 60 (73.17%)<br>UM: 5 (6.01%)	8	9.8%
Lesche (2020)	66	24 (36%)	Esomeprazole (9, 13%)<br>Citalopram (8, 12%)<br>Sertraline (5, 8%)<br>Fluoxetine (5, 8%)<br>Pantoprazole (4, 6%)	PM: 5 (7.58%)<br>IM: 20 (30.30%)<br>NM: 41 (62.12%)	PM: 9 (13.64%)<br>IM: 21 (31.82%)<br>NM: 36 (7.57%)	5	7.6%

			Oral contraceptive (2, 3%)				
Hole (2021)	93	59 on 150 mg/d Bupropion	Bupropion (93, 30.39%)	-	-	11	19.6%
		34 on ≥300 mg/d Bupropion		-	-	17	50.0%
Mostafa (2021)	137	-	Sertraline (15, 11%)<br>Duloxetine (11, 8%)<br>Fluoxetine (2, 1%)<br>Paroxetine (2, 1%)<br>Terbinafine (1, 0.7%)	PM: 6 (4.4%)<br>IM: 48 (35.0%)<br>NM: 77 (56.2%)<br>UM: 6 (4.4%)	PM: 10 (7.3%)<br>IM: 64 (46.7%)<br>NM: 57 (41.6%)<br>UM: 6 (4.4%)	20	14.6%
Jallaq (2021)	277	216 (77.98%)	Bupropion (71, 32.9%)<br>Fluoxetine (67, 31%)<br>Sertraline (58, 26.9%)<br>Duloxetine (18, 5.7%)<br>Fluvoxamine (2,1%)	PM: 14 (5.05%)<br>IM: 101 (36.5%)<br>NM: 158 (57%)<br>UM: 4 (1.4%)	PM: 134 (48.4%)<br>IM: 67 (24.2%)<br>NM: 75 (27.1%)<br>UM: 1 (0.36%)	120	43.3%
Gloor (2022)[2]	60	33 (55.0%)	-	PM: 1.7%<br>IM: 35.0%<br>NM: 56.7%<br>UM: 6.7%	-	10	16.7%
den Uil (2023)	117	26 (22.22%)	-	PM: 6.8%<br>IM: 36.8%<br>NM: 52.1%<br>UM: 4.3%	PM: 16.2%<br>IM: 32.5%<br>NM: 47.0%<br>UM: 4.3%	11	9.4%
Scherf-Clavel (2023)	316	-	-	PM: 4.9%<br>IM: 37.8%<br>NM: 55.1%<br>UM: 2.2%	PM: 12.6%<br>IM: 41.7%<br>NM: 44.8%<br>UM: 0.9%	36	11.6%
Tonti (2024)	193	29 (15%)	-	PM: 12 (6.2%)<br>IM: 74 (38.3%)<br>NM: 104 (53.9%)<br>UM: 3 (1.6%)	PM: 37 (19.2%)<br>IM: 63 (32.6%)<br>NM: 92 (47.7%)<br>UM: 1 (0.5%)	25	13.0%
Patel (2024)	4 114	1 980 (48%)	Most common were:<br>Bupropion (1 002, 24.36%)<br>Fluoxetine (952, 23.14%)	-	-	1 734	42.2%
Kanu (2024)	263 on risperidone	-	-	-	PM: 87 (33.1%)<br>IM: 75 (28.5%)<br>NM: 78 (29.7%)<br>UM: 23 (8.7%)	16	6.1%

	124 on paliperidone				PM: 53 (42.7%)<br>IM: 23 (18.5%)<br>NM: 36 (29.0%)<br>UM: 7 (5.6%)	23	8.7%
Holck (2024)	147	34 (24.82%)	Bupropion (20, 13.6%)<br>Paroxetine (9, 6.1%<br>Duloxetine (8, 5.4%)<br>Fluoxetine (2, 1.4%)	-	-	27	18.4%
De Brabander (2024)	412	102 (24.76%)	Paroxetine (60, 47.2%),<br>Citalopram (28, 22%),<br>Fluoxetine (14, 11%),<br>Sertraline (13, 10.2%),<br>Escitalopram (7, 5.5%),<br>Clomipramine (4, 3.1%)<br>Levomepromazine (1, 0.8%)	PM: 16 (4.7%)<br>IM 130 (38%)<br>NM: 196 (57.3%)	PM: 67 (19.6%)<br>IM 101 (29.5%)<br>NM: 174 (50.9%)	65	15.8%
Gerlach (2024)	214	40 (18.7%)	Fluoxetine (33, 15.4%)<br>Bupropion (7, 3.2%)<br>Duloxetine (1, 0.5%)	PM: 13 (6.1%)<br>IM: 88 (41.1%)<br>NM: 109 (50.9%)<br>UM: 4 (1.9%)	PM: 47 (22.0%)<br>IM: 77 (36.0%)<br>NM: 86 (40.2%)<br>UM: 4 (1.9%)	34	15.9%
De Brabander (2025)	708	-	Paroxetine (83, 42.15%)<br>Citalopram (44, 22.3%)<br>Fluoxetine (25, 12.7%)<br>Sertraline (12, 11.2%)<br>Escitalopram (11, 5.6%)<br>Clomipramine (8, 4.1%)<br>Levomepromazine (3, 1.5%)	PM: 39 (5.5%)<br>IM: 284 (40.1%)<br>NM: 375 (53%)<br>Unknown: 10 (1.4%)	-	95	13.4%
Gerlach (2025)	1 281	313 (24.4%)	Fluoxetine (257, 20.1%)<br>Bupropion (39, 3.0%)<br>Clobazam (11, 0.9%)<br>Duloxetine (11, 0.9%)<br>Doxepin (7, 0.5%)<br>Paroxetine (3, 0.2%)	PM: 6%<br>IM: 41%<br>NM: 51%<br>UM: 2%	PM: 27%<br>IM: 34%<br>NM: 37%<br>UM: 2%	269	21.0%
Gras (2025)	466	-		PM: 19 (4.1%)<br>IM: 142 (30.5%)<br>NM: 284 (60.9%)<br>UM: 21 (4.5%)	PM: 52 (11.2%)<br>IM: 139 (29.8%)<br>NM: 255 (54.7%)<br>UM: 20 (4.3%)	33	7.1%

PM Poor Metaboliser, *IM* Intermediate Metaboliser, *NM* Normal Metaboliser, *UM* Ultrarapid Metaboliser

Prevalence of CYP2D6 phenoconversion reported among individuals with a psychiatric disorder. All instances of phenoconversion were due to the use of either a strong or moderate CYP2D6 inhibitor.

[1] Shaded cells represent number of individuals/percentage of individuals who underwent CYP2D6 phenoconversion that were estimated using net change between genotype-predicted CYP2D6 phenotype and phenoconversion-adjusted CYP2D6 phenotype or where number of individuals were estimated when only percentage was given (Preskorn et al).

[2] Data for Gloor 2022 was taken from supplementary material s6 'potential drug induced pheno-conversion' using tutorial by Cicali et al to be comparable to other studies using Borges's standard multiplication factor and Flockhart table as source of inhibitors.

Table S13: JBI checklist for prevalence studies results

	Question	De Brabander 2024	De Brabander 2025	den Uil 2023	Gerlach 2024	Gerlach 2025	Gloor 2022	Gras 2025	Holck 2024	Hole 2021	Jallaq 2021	Kanu 2024	Lesche 2020	Mostafa 2021	Patel 2024	Patteet 2015	Preskorn 2013	Scherf-Clavel 2023	Tonti 2024
1	Was the sample frame appropriate to address the target population?	Yes	Yes	Yes	Yes	Yes	Yes	Yes	Yes	Yes	Yes	Yes	Yes	Yes	Yes	Yes	Yes	Yes	Yes
2	Were study participants recruited in an appropriate way to estimate phenoconversion in this population?	Yes	Yes	Yes	Yes	Yes	Yes	Yes	Yes	Yes	Yes	Yes	Yes	Yes	Yes	Yes	Yes	Yes	Yes
3	Was the sample size adequate?	Yes	Yes	No	Yes	Yes	No	Yes	Yes	No	Yes	Yes	No	No	Yes	No	Yes	Yes	Yes
4	Were the study subjects and setting described in detail?	Yes	Yes	Yes	Yes	Yes	Yes	Yes	Yes	Yes	Yes	Yes	Yes	Yes	Yes	Yes	Yes	Yes	Yes
5	Was data analysis conducted with sufficient coverage of the identified sample?	N/A	N/A	N/A	N/A	N/A	N/A	N/A	N/A	N/A	N/A	N/A	N/A	N/A	N/A	N/A	N/A	N/A	N/A
6	Were valid methods used for the identification of the condition?	Yes	Yes	Yes	Yes	Yes	Yes	Yes	Yes	Yes	Yes	Yes	Yes	Yes	Yes	Yes	Yes	Yes	Yes
7	Was the condition measured in a standard, reliable way for all participants?	Yes	Yes	Yes	Yes	Yes	Yes	Yes	Yes	Yes	Yes	No	Yes	Yes	Yes	Yes	Yes	Yes	Yes
8	Was there appropriate statistical analysis?	Yes	Yes	Yes	Yes	Yes	Yes	Yes	Yes	Yes	Yes	Yes	Yes	Yes	Yes	Yes	Yes	Yes	No
9	Was the response rate adequate, and if not, was the low response rate managed appropriately?	N/A	N/A	N/A	N/A	N/A	N/A	N/A	N/A	N/A	N/A	N/A	N/A	N/A	N/A	N/A	N/A	N/A	N/A
	Include?	Yes	Yes	Yes	Yes	Yes	Yes	Yes	Yes	Yes	Yes	Yes	Yes	Yes	Yes	Yes	Yes	Yes	Yes

Table S14: Leave-one-out analysis of CYP2D6 phenoconversion prevalence among individuals with a psychiatric disorder using the logit transformation

Study	Proportion	CI_lower	CI_upper	tau2	tau	I2
Omitting De Brabander 2025	-1.55915	-2.00952	-1.10879	0.51650	0.71868	0.97599
Omitting den Uil 2023	-1.52636	-1.95690	-1.09582	0.47511	0.68928	0.98056
Omitting Gerlach 2025	-1.61397	-2.06729	-1.16065	0.52323	0.72335	0.97701
Omitting Gloor 2022	-1.58527	-2.03302	-1.13753	0.51939	0.72068	0.98122
Omitting Holck 2024	-1.59650	-2.04903	-1.14396	0.52541	0.72485	0.98095
Omitting Jallaq 2021	-1.70087	-2.07189	-1.32985	0.33276	0.57686	0.98106
Omitting Lesche 2020	-1.51936	-1.94028	-1.09844	0.45912	0.67758	0.98093
Omitting Mostafa 2021	-1.57077	-2.02053	-1.12102	0.51953	0.72078	0.98074
Omitting Patel 2024	-1.70163	-2.07275	-1.33051	0.33132	0.57561	0.93141
Omitting Patteet 2015	-1.53396	-1.96635	-1.10157	0.48236	0.69452	0.98086
Omitting Scherf-clavel 2023	-1.54061	-1.98274	-1.09849	0.49766	0.70545	0.97908
Omitting Tonti 2024	-1.55679	-2.00392	-1.10965	0.51170	0.71533	0.98025

CI Confidence Intervals

Leave-one-out sensitivity analysis showing pooled prevalence estimates and heterogeneity measures following sequential omission of each study.

Table S15: Comparison of included and excluded participants by key characteristics

excluded	n	pct_phenoconverted	median_phq4	mean_age	pct_female
FALSE	30824	16.9	3	55.5	64
TRUE	281	15.3	4	55.2	60.1

Participants who had complete modified PHQ-4 scores from UK Biobank but were excluded due to missing data on ethnicity (n = 108), Townsend deprivation index (n = 48), smoking status (n = 80), or alcohol use (n = 48).

	level	Overall	Non-phenoconverted	Phenoconverted	p
n		30824	25612	5212	
Modified PHQ-4 score (mean (SD))		3.72 (3.16)	3.63 (3.15)	4.14 (3.18)	<0.001
Modified PHQ-4 score binary (%)	Modified PHQ-4 score <6	23501 (76.2)	19729 (77.0)	3772 (72.4)	<0.001
	Modified PHQ-4 score ≥6	7323 (23.8)	5883 (23.0)	1440 (27.6)	
Age (mean (SD))		55.46 (7.83)	55.51 (7.85)	55.21 (7.73)	0.012
Sex (%)	Female	19719 (64.0)	16086 (62.8)	3633 (69.7)	<0.001
	Male	11105 (36.0)	9526 (37.2)	1579 (30.3)	
Townsend deprivation (mean (SD))		-0.73 (3.36)	-0.73 (3.37)	-0.71 (3.29)	0.656
Number of self-reported medications (mean (SD))		4.97 (3.27)	4.81 (3.23)	5.72 (3.37)	<0.001
Number of self-reported indications (mean (SD))		3.56 (2.33)	3.54 (2.32)	3.68 (2.40)	<0.001
Smoking (%)	Never	14947 (48.5)	12566 (49.1)	2381 (45.7)	<0.001
	Current	5230 (17.0)	4313 (16.8)	917 (17.6)	
	Previous	10647 (34.5)	8733 (34.1)	1914 (36.7)	
Alcohol (%)	Once or twice a week	6804 (22.1)	5661 (22.1)	1143 (21.9)	0.007
	Daily or almost daily	6027 (19.6)	5006 (19.5)	1021 (19.6)	
	Never	4016 (13.0)	3395 (13.3)	621 (11.9)	
	One to three times a month	3661 (11.9)	2975 (11.6)	686 (13.2)	
	Special occasions only	4612 (15.0)	3812 (14.9)	800 (15.3)	

	Three or four times a week	5704 (18.5)	4763 (18.6)	941 (18.1)	
Ethnicity binary (%)	British or Irish	28837 (93.6)	23925 (93.4)	4912 (94.2)	0.028
	Other	1987 (6.4)	1687 (6.6)	300 (5.8)	

Table S16: Characteristics of the modified PHQ-4 score analysis sample stratified by phenoconversion status

Baseline characteristics of the UK Biobank cohort with full modified PHQ-4 scores at instance 0 stratified by CYP2D6 phenoconversion status. Values are represented as a mean (standard deviation) or n (%).

Table S17: Negative binomial regression of modified PHQ-4 total score by CYP2D6 phenoconversion status

Term	IRR	95% CI	p-value
(Intercept)	8.74	8.14-9.4	0.00e+00
Phenoconverted	1.10	1.07-1.13	8.48e-14
CYP2D6 Intermediate Metabolizer	1.01	0.99-1.03	0.467
CYP2D6 Poor Metabolizer	1.01	0.97-1.05	0.543
CYP2D6 Ultrarapid Metabolizer	1.02	0.94-1.09	0.679
Age	0.98	0.98-0.98	6.49e-264
Male	1.05	1.03-1.07	1.03e-06
Ethnicity Other	1.08	1.04-1.12	7.71e-05
Townsend deprivation	1.03	1.02-1.03	4.25e-64
Number of self-reported medications	1.04	1.04-1.04	8.10e-111
Number of self-reported indications	1.02	1.02-1.03	3.28e-20
Smoking Current	1.17	1.14-1.2	1.66e-29
Smoking Previous	0.98	0.96-1	0.102
Alcohol Daily or almost daily	0.97	0.94-0.99	0.021
Alcohol Never	1.11	1.08-1.15	3.61e-10
Alcohol One to three times a month	1.06	1.02-1.09	0.002
Alcohol Special occasions only	1.12	1.09-1.16	6.31e-13
Alcohol Three or four times a week	0.94	0.91-0.97	3.94e-05

Results are reported as incidence rate ratios (IRR) with 95% confidence intervals. Reference categories: CYP2D6 Normal Metabolizer (genotypic phenotype); female (sex); White British or Irish (ethnicity); never smoked (smoking); once or twice a week (alcohol). Model adjusted for genotypic CYP2D6 phenotype, age, sex, ethnicity, Townsend deprivation index, number of self-reported medications, number of self-reported indications, smoking status, and alcohol use. Modified PHQ-4 scores exhibited overdispersion (mean = 3.72, variance = 10.00), justifying the use of negative binomial over Poisson regression.

Table S18: CYP2D6 substrate-inhibitor interaction table

					Drug Interaction Potential		
**Drugs metabolised by CYP2D6**	**Clinical uses of drug**	**Extent of the role of CYP2D6 in metabolism**	**Is the drug also a CYP2D6 inhibitor?**	**Examples of effects of co-prescribing drug(s) metabolised by CYP2D6 with a CYP2D6 inhibitor**	**Strong CYP2D6 Inhibitors**	**Moderate CYP2D6 Inhibitors**	**Weak CYP2D6 Inhibitors**
**Antidepressants** ~ *Depression, anxiety*							
Desipramine	Licensed: MDD<br>Off label: bulimia nervosa, IBS, neuropathic pain, overactive bladder, postherpetic neuralgia, ADHD	Major role		-			
Doxepin	Licensed: MDD, insomnia, anxiety, pruritus<br>Off label: neuropathic pain, migraines, urethral irritation/dysuria	Major role		• Increased sedation and a drug-induced fall reported when co-administered with the strong CYP2D6 inhibitor fluoxetine.[47]			
Fluoxetine	Licensed: MDD, OCD, panic disorder, bulimia nervosa, TRD<br>Off label: binge eating disorder, SAD, PMDD, borderline personality disorder, PTSD, Raynaud phenomenon, selective mutism	Major role	Strong inhibitor	• Increased sedation and a drug-induced fall reported when co-administered with the moderate CYP2D6 inhibitor doxepin.[47]			
Nortriptyline	Licensed: MDD<br>Off label: chronic pain, diabetic neuropathy, myofascial pain, trigeminal neuralgia,	Major role		• Adding 5mg of the strong CYP2D6 inhibitor paroxetine to patients with high hydroxynortriptyline levels (CYP2D6 ultrarapid metabolisers) can provide safer treatment with nortriptyline.[48]			

	postherpetic neuralgia, migraine prophylaxis, smoking cessation, neurogenic cough,						
Paroxetine	Licensed: MDD, OCD, SAD, PTSD, GAD, PMDD, vasomotor symptoms associated with menopause<br>Off label: OCD/SAD (paediatric), separation anxiety, dysthymia, body dysmorphic disorder, postpartum depression, premature ejaculation	Major role	Strong inhibitor	• Co-administration with metoprolol resulted in ADRs including bradycardia, hypotension and drowsiness.[49–51]<br>• In a suicide attempt a man ingested fifty 20mg paroxetine tablets resulting in extremely long intoxication accompanied by delirium.[52]			
Venlafaxine	Licensed: MDD, SAD, panic disorder<br>Off label: ADHD, fibromyalgia, complex pain syndromes, cataplexy, hot flashes, PTSD, OCD, PMDD, migraine prophylaxis, diabetic neuropathy	Major role		• Co-administration with both CYP2D6 and CYP2C19 inhibitors resulted in various ADRs and treatment inefficacy.[53]			
Duloxetine	Licensed: MDD, GAD, fibromyalgia, chronic musculoskeletal pain, diabetic peripheral neuropathy<br>Off label: chemotherapy-induced peripheral neuropathy, stress urinary incontinence	Partial role	Moderate inhibitor	• Duloxetine-mediated CYP2D6 phenoconversion, spanning 10 years, resulted in only minor improvement of MDD symptoms and uncontrolled pain despite use of a multimodal analgesic regimen.[54]			

Escitalopram	Licensed: MDD, GAD<br>Off label: SAD, OCD, panic disorder, PTSD, PMDD, menopausal vasomotor symptoms	Partial role	Weak inhibitor	• Co-administration with both CYP2D6 and CYP2C19 inhibitors resulted in various ADRs and treatment inefficacy.[53]			
Fluvoxamine	Licensed: OCD<br>Off label: MDD, GAD, bulimia nervosa, PMDD, PTSD, panic disorder, separation anxiety disorder (paediatric)	Partial role	Weak inhibitor	-			
Imipramine	Licensed: MDD, nocturnal enuresis (paediatric)<br>Off label: chronic neuropathic pain, panic disorder	Minor role		-			
**Antiarrhythmics** ~ *Abnormal heart rhythms*							
Metoprolol	Licensed: hypertension, angina, heart failure, myocardial infarction<br>Off label: supraventricular tachycardia, ventricular tachycardia, migraine prophylaxis, essential tremor	Major role		• Co-administration with the strong CYP2D6 inhibitor paroxetine, resulted in ADRs including bradycardia, hypotension and drowsiness.[49–51]			
Nebivolol	Licensed: hypertension<br>Off label: heart failure, microvascular angina, cancer therapy-related cardiac dysfunction	Major role		-			
Encainide	No longer prescribed	Major role		-			

Mexiletine	<u>Licensed</u>: ventricular arrhythmias<br><u>Off label</u>: peripheral neuropathy, chronic pain, long QT syndrome (types 2 & 3), myotonic dystrophy	Partial role		• Co-administration with the strong CYP2D6 inhibitor quinidine lead to improved efficacy at smaller doses of mexiletine and decreased side-effects (gastric irritation).[55]			
Flecainide	<u>Licensed</u>: supraventricular tachycardia, Wolff-Parkinson-White syndrome, atrial fibrillation/flutter (no structural heart disease), ventricular arrhythmias<br><u>Off label</u>: long QT syndrome type 3, catecholaminergic polymorphic ventricular tachycardia, Andersen-Tawil syndrome type 1	Minor role		-			
**Analgesics** ~ *Pain*							
Codeine	<u>Licensed</u>: mild-moderate pain<br><u>Off label</u>: chronic cough, restless leg syndrome, persistent diarrhoea (palliative)	Major role		• Genotypic NMs on CYP2D6-metabolised opioids + a strong/moderate CYP2D6 inhibitor had 49% more pain-related ED visits compared to those not taking an inhibitor.[56]<br>• Reduced CYP2D6 activity due to inhibitor use was associated with more uncontrolled pain when co-prescribed with codeine or tramadol.[57]			
Tramadol	<u>Licensed</u>: moderate-severe pain<br><u>Off label</u>: premature ejaculation, restless leg syndrome	Major role		• Genotypic NMs on CYP2D6-metabolised opioids + strong/moderate CYP2D6 inhibitor had 49% more pain-related ED visits compared to those not taking an inhibitor.[56]			

				• Co-administration with the moderate CYP2D6 inhibitor duloxetine resulted in loss of analgesic effect.[58]<br>• Reduced CYP2D6 activity due to inhibitor use was associated with more uncontrolled pain when co-prescribed with codeine or tramadol.[57]			
Oxycodone	<u>Licensed</u>: moderate-severe pain (acute and chronic)<br><u>Off label</u>: restless leg syndrome, dyspnoea	Partial role		• Genotypic NMs on CYP2D6-metabolised opioids + strong/moderate CYP2D6 inhibitor had 49% more pain-related ED visits compared to those not taking an inhibitor.[56]<br>• Co-administration with the strong CYP2D6 inhibitor paroxetine resulted in loss of analgesic effect.[59]			
**Antipsychotics** ~ *Schizophrenia, Bipolar disorder*							
Haloperidol	<u>Licensed</u>: schizophrenia, Tourette syndrome, severe behavioural disorders (paediatric), hyperactivity (paediatric)<br><u>Off label:</u> agitation, acute mania, chemotherapy-induced nausea and vomiting, intractable hiccups	Partial role	In-vitro evidence only	-			
**Antitussives** ~ *Cough suppressant*							
Dextromethorphan	<u>Licensed</u>: cough (antitussive), pseudobulbar affect (with quinidine), MDD (with bupropion)<br><u>Off label:</u> stroke, traumatic brain injury, refractory seizures, analgesia, methotrexate	Major role		-			

	neurotoxicity, Parkinson's disease, autism, pulmonary fibrosis						
**Illicit stimulants**							
Methoxyamphetamine	No licensed or off-label clinical use	Partial role		-			
Mephedrone	No licensed or off-label clinical use	Partial role		-			
**VMAT 2 Inhibitors** ~ *Tardive dyskinesia, Huntington's disease, Tourette syndrome*							
Deutetrabenazine	Licensed: Tardive dyskinesia, Huntington's disease<br>Off label: Tourette syndrome	Major role		-			
Tetrabenazine	Licensed: Huntington's disease, moderate to severe tardive dyskinesia<br>Off label: hemiballismus, Tourette syndrome, other hyperkinetic movement disorders	Major role		-			
**Acetylcholinesterase inhibitors** ~ *Alzheimer's disease*							
Donepezil	Licensed: Alzheimer's disease<br>Off label: Lewy body dementia, traumatic brain injury, vascular dementia, Parkinson's disease dementia	Minor role		-			
**Anorexiants/appetite suppressants** ~ *Obesity*							
Dexfenfluramine	No longer prescribed	Minor role		-			
**Antihypertensives** ~ *High blood pressure*							
Debrisoquine	No longer prescribed	Major role		-			

**Glucosylceramide synthase inhibitors** ~ *Type 1 Gaucher disease*							
Eliglustat	Licensed: Type 1 Gaucher disease<br>Off label: -	Major role		-			
**Antianginals** ~ *Chest pain*							
Perhexiline	Licensed: severe angina pectoris<br>Off label: intractable angina, heart failure with normal left ventricular systolic contraction and hypertrophic cardiomyopathy	Major role		-			
**Antiemetics** ~ *Prevent/treat nausea and vomiting*							
S-Ondansetron	Licensed: chemotherapy-induced nausea and vomiting, radiation-induced nausea and vomiting, postoperative nausea and vomiting<br>Off label: pregnancy-related nausea and vomiting, cyclic vomiting syndrome (paediatric), carcinoid syndrome diarrhoea	Major role		-			
Metoclopramide	Licensed: GERD-associated nausea and vomiting, diabetic gastroparesis, chemotherapy-induced nausea and vomiting<br>Off label: postoperative nausea and vomiting,	Partial role		-			

	acute migraine, hyperemesis gravidarum, Diamond Blackfan syndrome, nausea in advanced liver disease						

Colour	Inhibitor-substrate interaction potential
	High
	Moderate
	Low

ADR Adverse Drug Reaction, *MDD* Major Depressive Disorder, *NM* Normal Metaboliser, *ED* Emergency Department, *IBS* Irritable Bowel Syndrome, *ADHD* Attention-Deficit/Hyperactivity Disorder, *OCD* Obsessive-Compulsive Disorder, *TRD* Treatment-Resistant Depression, *SAD* Social Anxiety Disorder, *PMDD* Premenstrual Dysphoric Disorder, *GAD* Generalised Anxiety Disorder, *GERD* Gastroesophageal Reflux Disease

PUBMED was searched for the terms "phenoconversion" AND each CYP2D6 substrate (e.g. "paroxetine") to find examples of reported effects resulting from the prescription of a CYP2D6 substrate along with a CYP2D6 inhibitor. Licensed and off-label uses taken from StatPearls where applicable and off-label uses may not be exhaustive. List of CYP2D6 substrates, the extent to which CYP2D6 is involved in their metabolism and CYP2D6 inhibitor status were each taken from the Drug Interactions Flockhart Table.

Supplementary Figures

Figure S1: PRISMA flow diagram

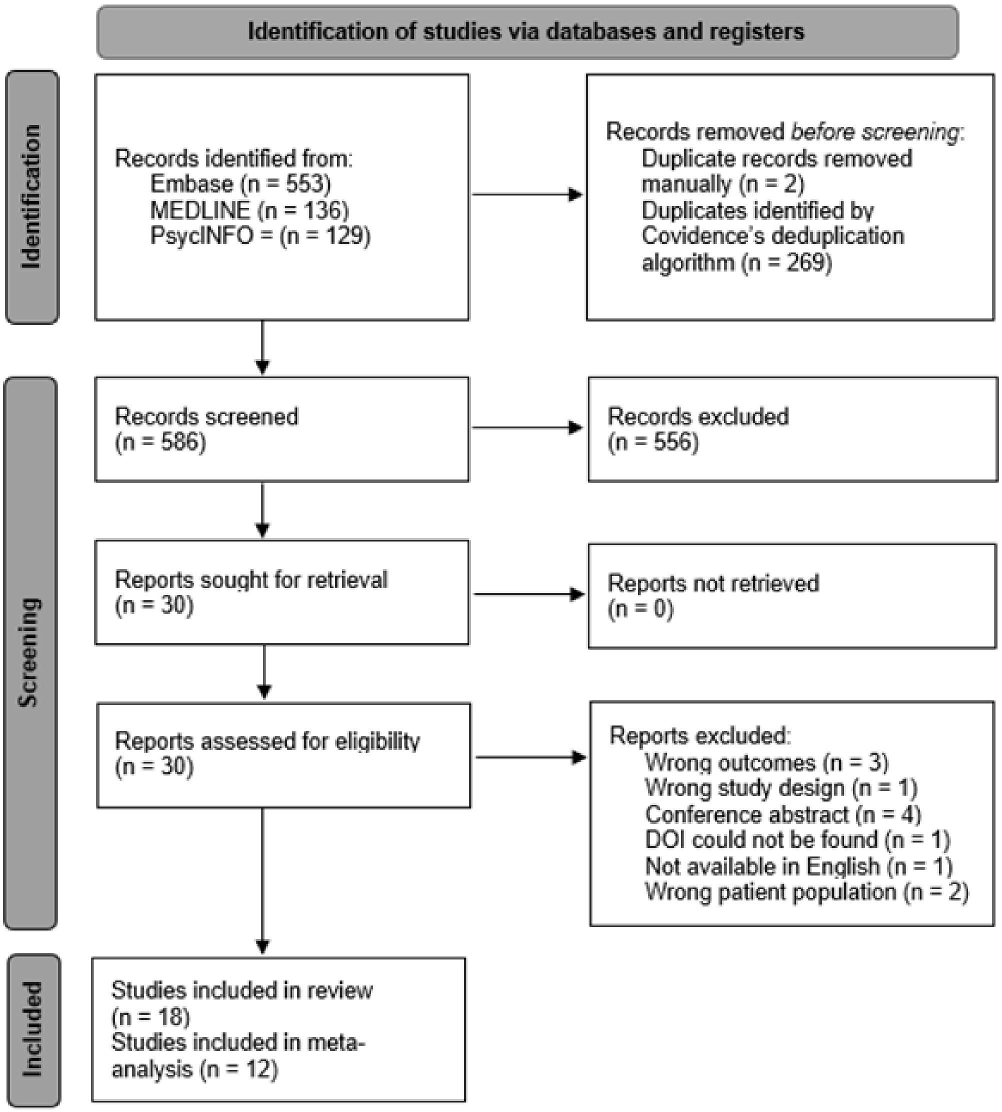

DOI Digital Object Identifier

Of the 818 unique identified studies, 30 full-text articles were assessed for eligibility, 18 were included in the final analysis and 12 studies in the meta-analysis.

Figure S2: Meta-analysis of CYP2D6 phenoconversion prevalence among individuals with a psychiatric disorder using the logit

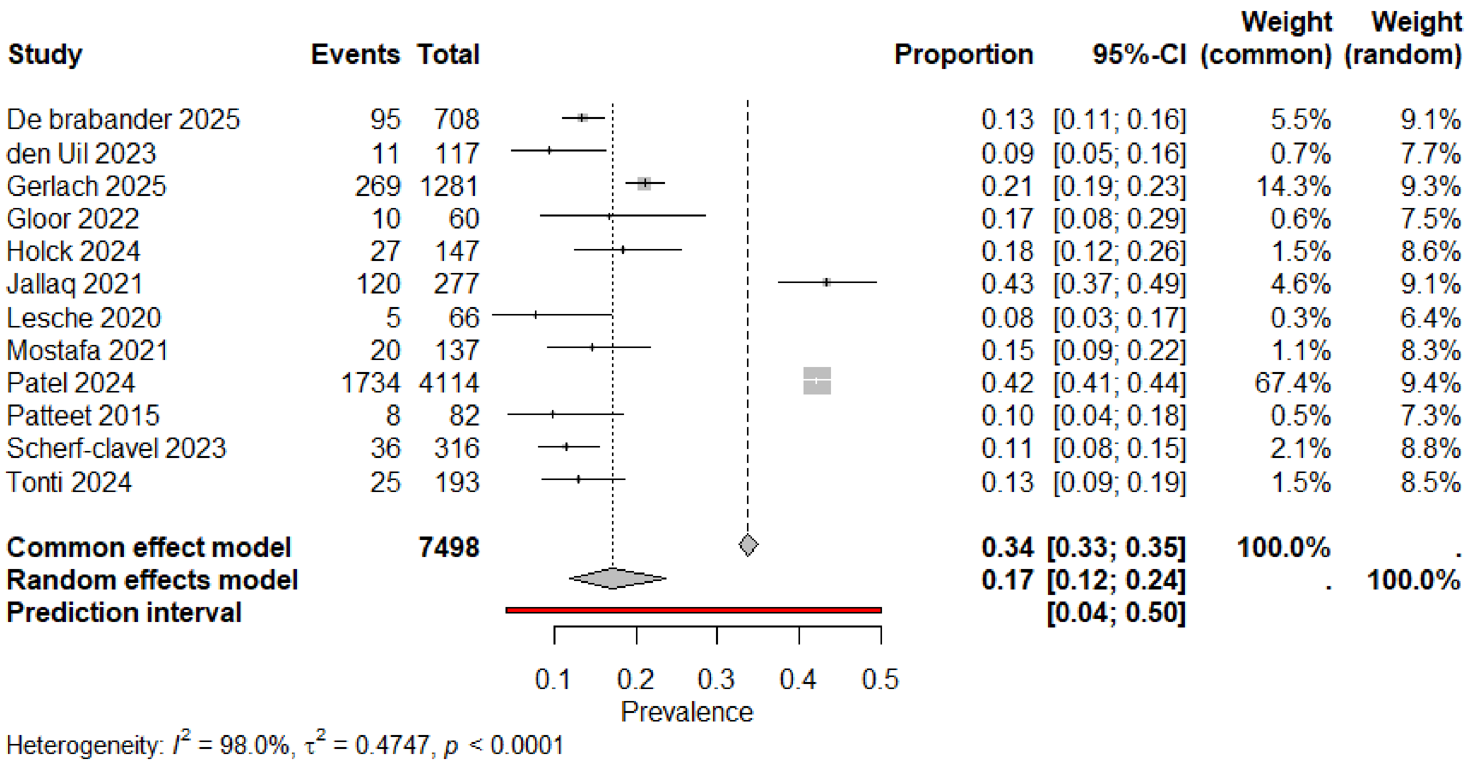

transformation

Forest plot of the pooled prevalence of CYP2D6 phenoconversion among individuals with a psychiatric disorder using a random effects model with the logit transformation. Squares represent individual study estimates weighted by study size with a horizontal line to represent the 95% confidence intervals (CIs) and the diamond represents the pooled prevalence and the 95% CIs. The red horizontal bar represents the prediction interval. Heterogeneity was assessed using the I^2 statistic ($I^2 = 98\%$).

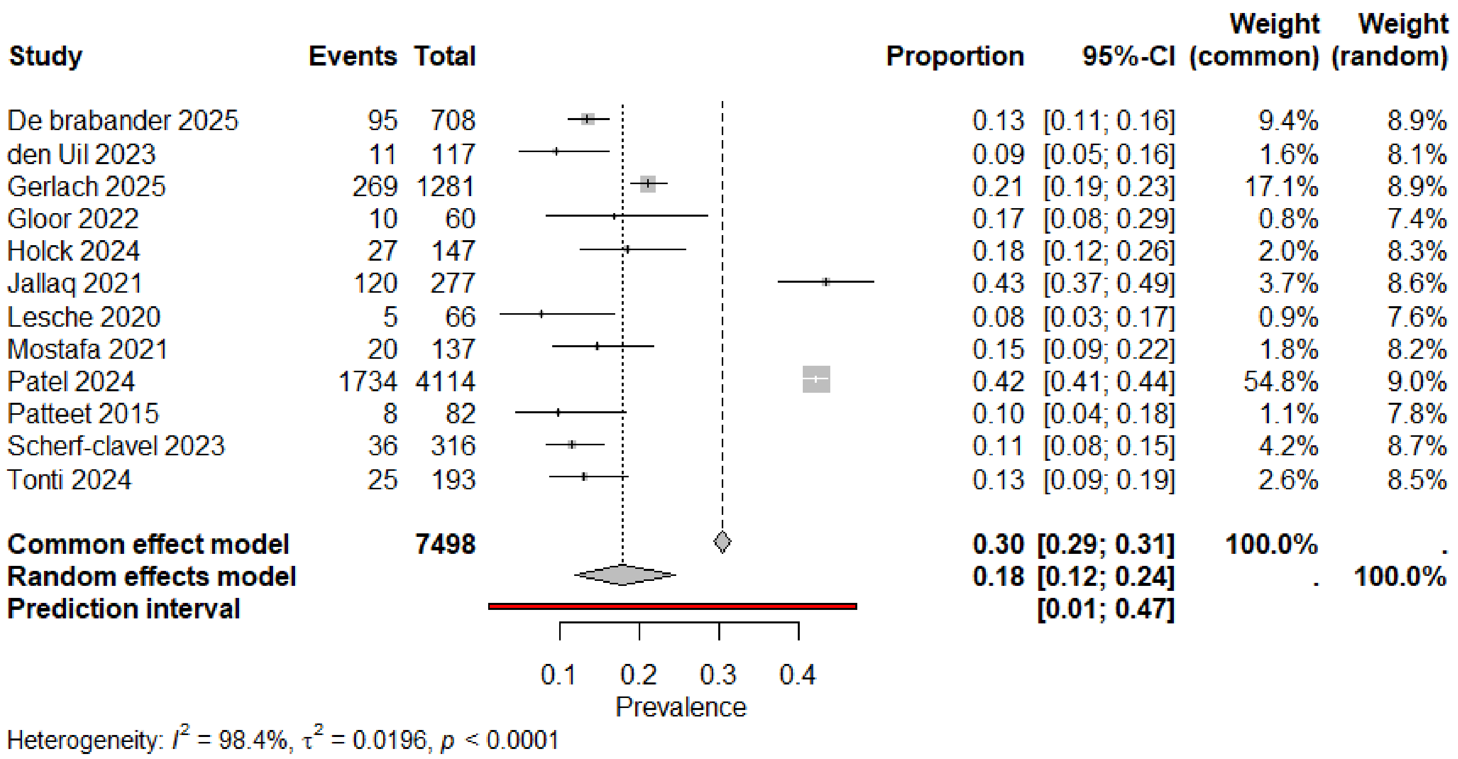

Figure S3: Meta-analysis of CYP2D6 phenoconversion prevalence among individuals with a psychiatric disorder using the Freeman-Tukey double arcsine transformation

Forest plot of the pooled prevalence of CYP2D6 phenoconversion among individuals with a psychiatric disorder using a random effects model with the Freeman-Tukey double arcsine transformation. Squares represent individual study estimates weighted by study size with a horizontal line to represent the 95% confidence intervals (CIs) and the diamond represents the pooled prevalence and the 95% CIs. The red horizontal bar represents the prediction interval. Heterogeneity was assessed using the I^2 statistic ($I^2 = 98\%$).

Figure S4: Doi plot of CYP2D6 phenoconversion prevalence among individuals with a psychiatric disorder using the logit transformation

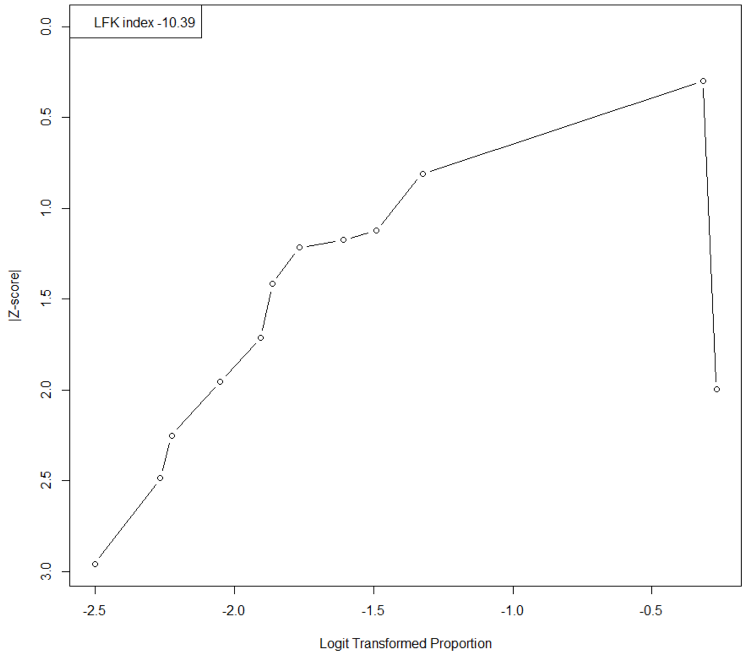

Doi plot assessing small study effects and publication bias. The Luis-Fuyura-Kanamori (LFK) index was -10.39, indicating major asymmetry.

Figure S5: Galbraith plot of CYP2D6 phenoconversion prevalence among individuals with a psychiatric disorder using the logit transformation

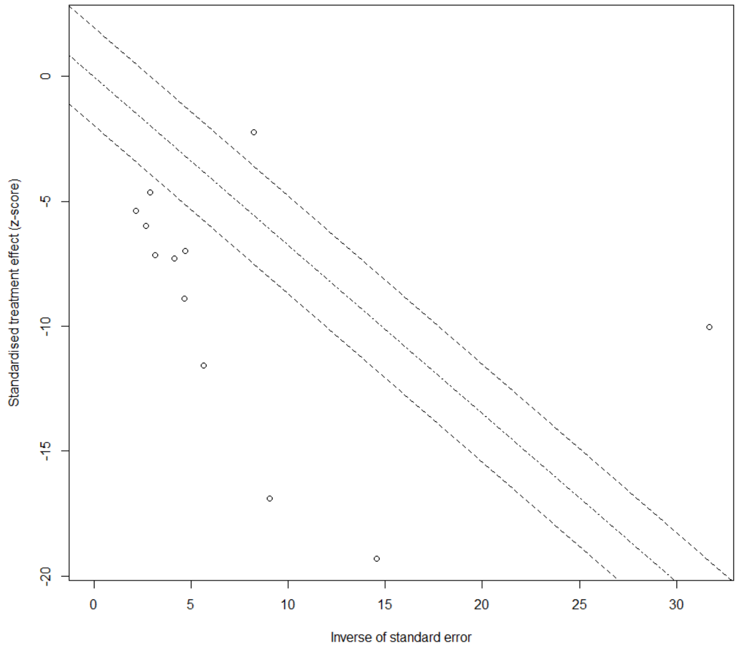

Galbraith plot assessing small study effects and publication bias displaying asymmetry.

Figure S6: Subgroup analysis of CYP2D6 phenoconversion prevalence among individuals with a psychiatric disorder using the logit transformation by age

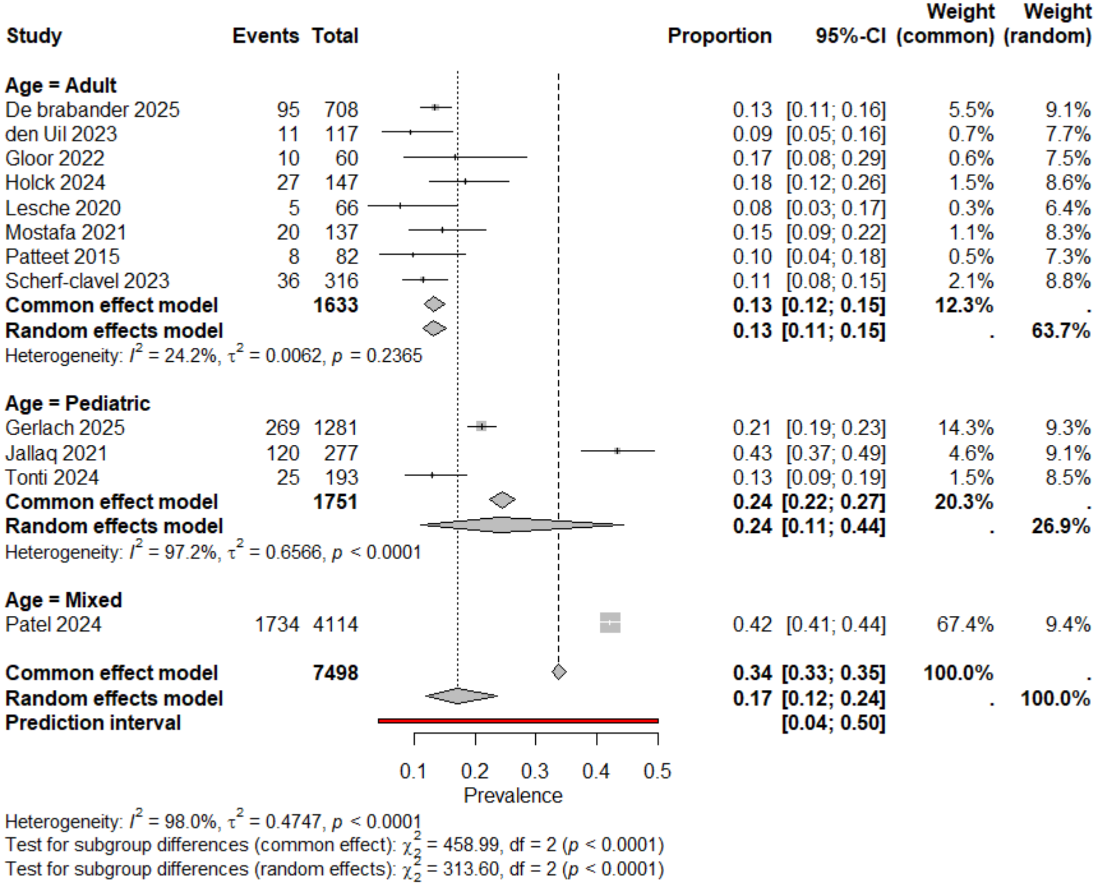

Subgroup analysis of the pooled prevalence of CYP2D6 phenoconversion among individuals with a psychiatric disorder using a random effects model with the logit transformation by age. Adult subgroup includes studies with a cohort exclusively ≥19 years old, paediatric <19 years old and mixed spanning both.

Squares represent individual study estimates weighted by study size with a horizontal line to represent the 95% confidence intervals (CIs) and the diamond represents the pooled prevalence and the 95% CIs. The red horizontal bar represents the prediction interval. Heterogeneity was assessed using the I^2 statistic (I^2 = 98%).

Figure S7: Meta-analysis of CYP2D6 phenoconversion prevalence among adults with a psychiatric disorder using the Freeman-Tukey double arcsine transformation

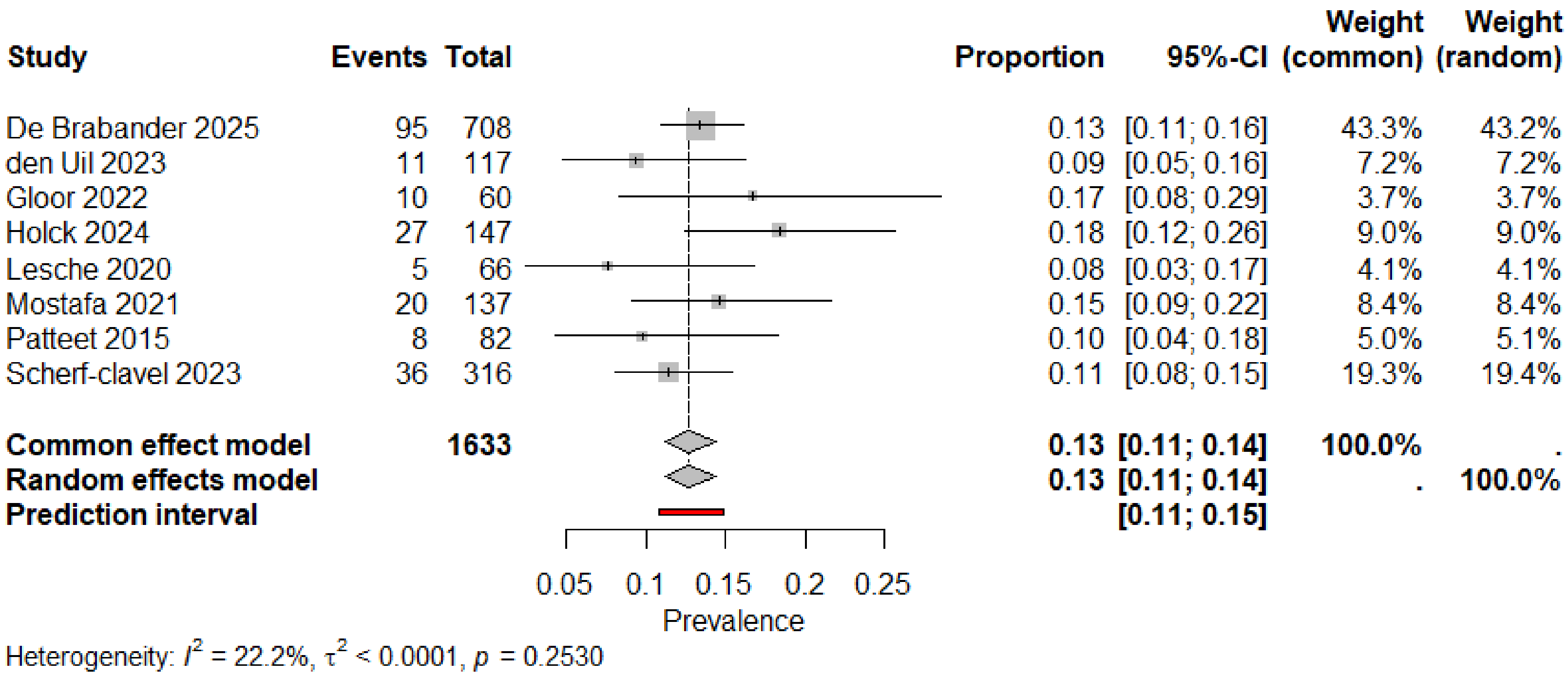

Forest plot of the pooled prevalence of CYP2D6 phenoconversion among adults with a psychiatric disorder using a random effects model with the Freeman-Tukey double arcsine transformation. Squares represent individual study estimates weighted by study size with a horizontal line to represent the 95% confidence intervals (CIs) and the diamond represents the pooled prevalence and the 95% CIs. The red horizontal bar represents the prediction interval. Heterogeneity was assessed using the I^2 statistic ($I^2 = 22.2\%$).

Figure S8: Doi plot of CYP2D6 phenoconversion prevalence among adults with a psychiatric disorder using the logit transformation

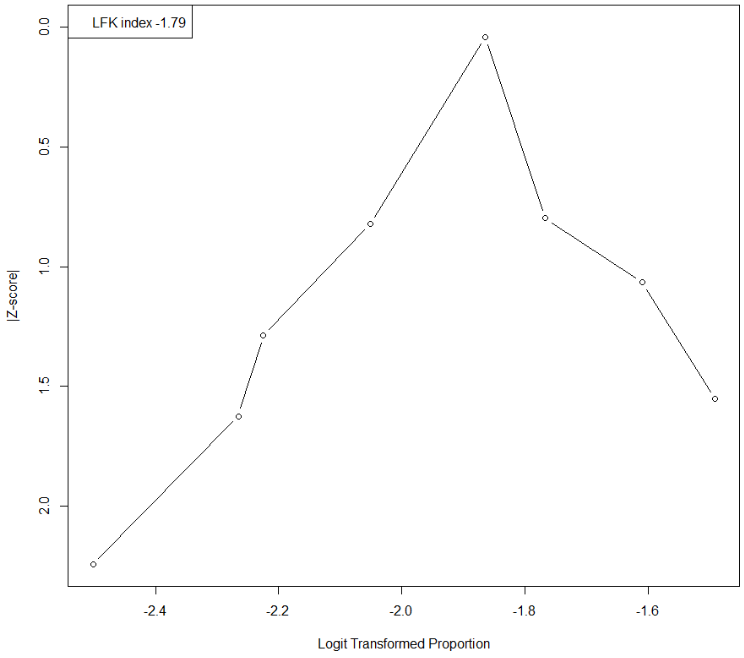

Doi plot assessing small study effects and publication bias. The Luis-Fuyura-Kanamori (LFK) index was -1.79, indicating minor asymmetry.

Figure S9: Galbraith plot of CYP2D6 phenoconversion prevalence among adults with a psychiatric disorder using the logit transformation

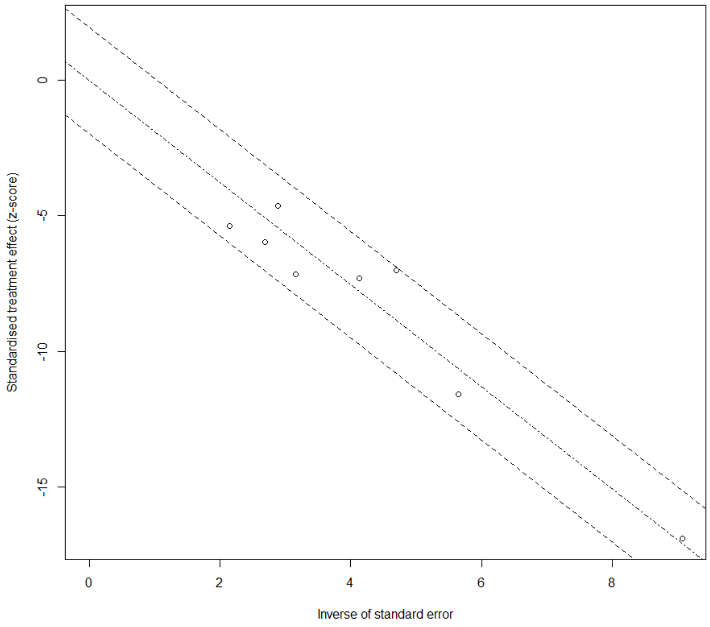

Galbraith plot assessing small study effects and publication bias displaying asymmetry.

Figure S10: Doi plot of CYP2D6 phenoconversion prevalence among individuals with a psychiatric disorder following removal of Patel et al.

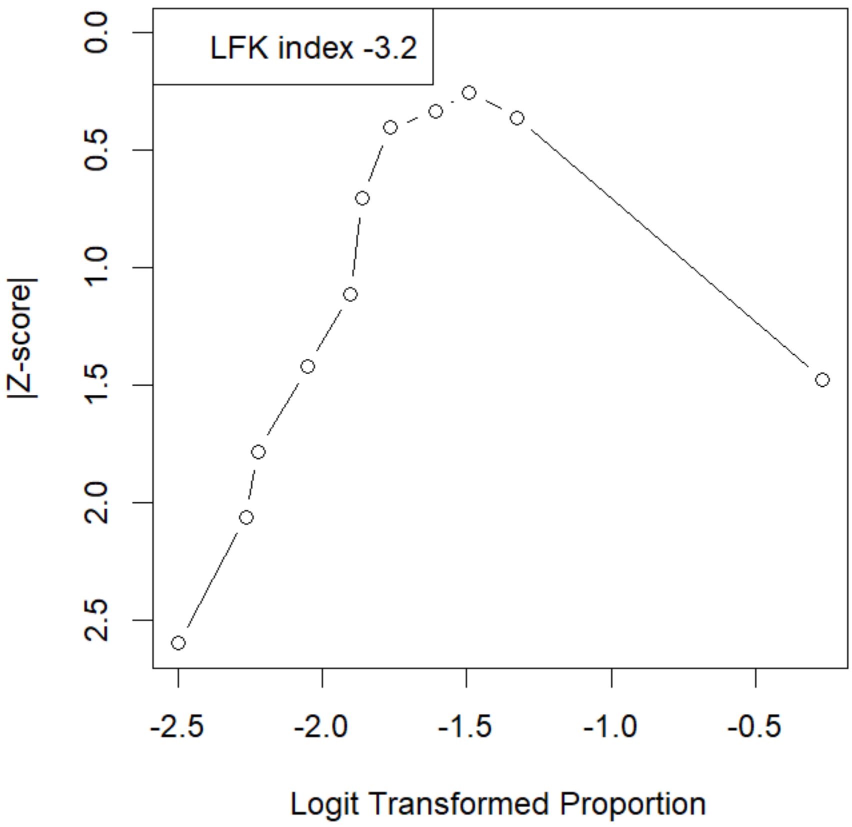

Doi plot assessing small study effects and publication bias following removal of Patel et al. The Luis-Fuyura-Kanamori (LFK) index was -3.2, indicating major asymmetry.

References

1. Flockhart, D.A.; Thacker, D.; McDonald, C.; Desta, Z. *The Flockhart Cytochrome P450 Drug-Drug Interaction Table*; **2021**.
2. P, A.T.; M, S.S.; Jose, A.; Chandran, L.; Zachariah, S.M. Pharmacogenomics: The Right Drug to the Right Person. *Journal of Clinical Medicine Research* **2009**, *1*, 191, doi:10.4021/jocmr2009.08.1255.
3. Zhao, M.; Ma, J.; Li, M.; Zhang, Y.; Jiang, B.; Zhao, X.; Huai, C.; Shen, L.; Zhang, N.; He, L.; et al. Cytochrome P450 Enzymes and Drug Metabolism in Humans. *Int J Mol Sci* **2021**, *22*, 12808, doi:10.3390/ijms222312808.
4. Klomp, S.D.; Manson, M.L.; Guchelaar, H.-J.; Swen, J.J. Phenoconversion of Cytochrome P450 Metabolism: A Systematic Review. *JCM* **2020**, *9*, 2890, doi:10.3390/jcm9092890.
5. Shah, R.R.; Smith, R.L. Addressing Phenoconversion: The Achilles' Heel of Personalized Medicine. *Brit J Clinical Pharma* **2015**, *79*, 222–240, doi:10.1111/bcp.12441.
6. Koopmans, A.B.; Braakman, M.H.; Vinkers, D.J.; Hoek, H.W.; Van Harten, P.N. Meta-Analysis of Probability Estimates of Worldwide Variation of CYP2D6 and CYP2C19. *Transl Psychiatry* **2021**, *11*, 141, doi:10.1038/s41398-020-01129-1.
7. Petrović, J.; Pešić, V.; Lauschke, V.M. Frequencies of Clinically Important CYP2C19 and CYP2D6 Alleles Are Graded across Europe. *Eur J Hum Genet* **2020**, *28*, 88–94, doi:10.1038/s41431-019-0480-8.
8. Samer, C.F.; Lorenzini, K.I.; Rollason, V.; Daali, Y.; Desmeules, J.A. Applications of CYP450 Testing in the Clinical Setting. *Mol Diagn Ther* **2013**, *17*, 165–184, doi:10.1007/s40291-013-0028-5.
9. *Drug Development and Drug Interactions: Table of Substrates, Inhibitors and Inducers*; **2023**.
10. Nahid, N.A.; Johnson, J.A. CYP2D6 Pharmacogenetics and Phenoconversion in Personalized Medicine. *Expert Opinion on Drug Metabolism & Toxicology* **2022**, *18*, 769–785, doi:10.1080/17425255.2022.2160317.
11. Munn, Z.; Moola, S.; Riitano, D.; Lisy, K. The Development of a Critical Appraisal Tool for Use in Systematic Reviews Addressing Questions of Prevalence. *Int J Health Policy Manag* **2014**, *3*, 123–128, doi:10.15171/ijhpm.2014.71.
12. Barendregt, J.J.; Doi, S.A.; Lee, Y.Y.; Norman, R.E.; Vos, T. Meta-Analysis of Prevalence. *J Epidemiol Community Health* **2013**, *67*, 974–978, doi:10.1136/jech-2013-203104.
13. Guyatt, G.H.; Oxman, A.D.; Vist, G.E.; Kunz, R.; Falck-Ytter, Y.; Alonso-Coello, P.; Schünemann, H.J. GRADE: An Emerging Consensus on Rating Quality of Evidence and Strength of Recommendations. *BMJ* **2008**, *336*, 924–926, doi:10.1136/bmj.39489.470347.AD.
14. Cicali, E.J.; Elchynski, A.L.; Cook, K.J.; Houder, J.T.; Thomas, C.D.; Smith, D.M.; Elsey, A.; Johnson, J.A.; Cavallari, L.H.; Wiisanen, K. How to Integrate CYP2D6 Phenoconversion Into Clinical Pharmacogenetics: A Tutorial. *Clin Pharma and Therapeutics* **2021**, *110*, 677–687, doi:10.1002/cpt.2354.
15. Naing, L.; Nordin, R.B.; Abdul Rahman, H.; Naing, Y.T. Sample Size Calculation for Prevalence Studies Using Scalex and ScalaR Calculators. *BMC Med Res Methodol* **2022**, *22*, 209, doi:10.1186/s12874-022-01694-7.
16. Patel, J.N.; Morris, S.A.; Torres, R.; Rhead, B.; Vlangos, C.; Mueller, D.J.; Brown, L.C.; Lefkofsky, H.; Ali, M.; De La Vega, F.M.; et al. Pharmacogenomic Insights in Psychiatric Care: Uncovering Novel Actionability, Allele-Specific CYP2D6 Copy Number Variation, and Phenoconversion in 15,000 Patients. *Mol Psychiatry* **2024**, *29*, 3495–3502, doi:10.1038/s41380-024-02588-4.
17. Den Uil, M.G.; Hut, H.W.; Wagelaar, K.R.; Abdullah-Koolmees, H.; Cahn, W.; Wilting, I.; Deneer, V.H.M. Pharmacogenetics and Phenoconversion: The Influence on Side Effects Experienced by Psychiatric Patients. *Front. Genet.* **2023**, *14*, 1249164, doi:10.3389/fgene.2023.1249164.
18. Caudle, K.E.; Sangkuhl, K.; Whirl-Carrillo, M.; Swen, J.J.; Haidar, C.E.; Klein, T.E.; Gammal, R.S.; Relling, M.V.; Scott, S.A.; Hertz, D.L.; et al. Standardizing CYP2D6 Genotype to Phenotype Translation: Consensus Recommendations from the Clinical Pharmacogenetics Implementation Consortium and Dutch Pharmacogenetics Working Group. *Clin Transl Sci* **2020**, *13*, 116–124, doi:10.1111/cts.12692.
19. Hole, K.; Arnestad, M.; Molden, E.; Haslemo, T. Dose-Dependent Inhibition of CYP2D6 by Bupropion in Patients With Depression. *J Clin Psychopharmacol* **2021**, *41*, 281–285, doi:10.1097/JCP.0000000000001387.

20. Preskorn, S.H.; Kane, C.P.; Lobello, K.; Nichols, A.I.; Fayyad, R.; Buckley, G.; Focht, K.; Guico-Pabia, C.J. Cytochrome P450 2D6 Phenoconversion Is Common in Patients Being Treated for Depression: Implications for Personalized Medicine. *J. Clin. Psychiatry* **2013**, *74*, 614–621, doi:10.4088/JCP.12m07807.
21. De Brabander, E.Y.; Leibold, N.K.; Van Amelsvoort, T.; Van Westrhenen, R.; Alizadeh, B.Z.; Cahn, W.; De Haan, L.; Van Der Pluijm, M.; Simons, C.J.P.; Van Os, J.; et al. Sex and Age Effects on Prevalence of CYP2C19 and CYP2D6 Phenoconversion Risk over Time in Patients with Psychosis. *Progress in Neuro-Psychopharmacology and Biological Psychiatry* **2025**, *138*, 111363, doi:10.1016/j.pnpbp.2025.111363.
22. Gras, C.; Piras, M.; Ranjbar, S.; Grosu, C.; Girardin, F.R.; Vandenberghe, F.; Ansermot, N.; Grandjean, C.; Kaiser, S.; Gamma, F.; et al. Influence of C *YP2D6* Genotypes and Phenotypes on the Plasma Levels and Clinical Response to Aripiprazole. *Schizophrenia Bulletin* **2025**, sbaf076, doi:10.1093/schbul/sbaf076.
23. Gloor, Y.; Lloret-Linares, C.; Bosilkovska, M.; Perroud, N.; Richard-Lepouriel, H.; Aubry, J.-M.; Daali, Y.; Desmeules, J.A.; Besson, M. Drug Metabolic Enzyme Genotype-Phenotype Discrepancy: High Phenoconversion Rate in Patients Treated with Antidepressants. *Biomedicine & Pharmacotherapy* **2022**, *152*, 113202, doi:10.1016/j.biopha.2022.113202.
24. Munn, Z.; Moola, S.; Lisy, K.; Riitano, D.; Tufanaru, C. Methodological Guidance for Systematic Reviews of Observational Epidemiological Studies Reporting Prevalence and Incidence Data. **2015**, *13*, 147–153.
25. De Brabander, E.Y.; Breddels, E.; Van Amelsvoort, T.; Van Westrhenen, R.; GROUP Investigators Clinical Effects of CYP2D6 Phenoconversion in Patients with Psychosis. *J Psychopharmacol* **2024**, *38*, 1095–1110, doi:10.1177/02698811241278844.
26. Holck, A.; Asp, M.; Green, H.; Westrin, Å.; Reis, M. CYP2D6 Genotyping and Inhibition as Predictors of Adverse Drug Reactions in Depressive Disorders. *J. Clin. Psychiatry* **2024**, *85*, doi:10.4088/JCP.23m14937.
27. Kanu, A.A.; Johnston, M.M.; Poweleit, E.A.; Vaughn, S.E.; Strawn, J.R.; Ramsey, L.B. Influence of CYP2D6 Metabolizer Status on Risperidone and Paliperidone Tolerability in Children and Adolescents. *Journal of Child and Adolescent Psychopharmacology* **2024**, *34*, 34–41, doi:10.1089/cap.2023.0046.
28. Lisbeth, P.; Vincent, H.; Kristof, M.; Bernard, S.; Manuel, M.; Hugo, N. Genotype and Co-Medication Dependent CYP2D6 Metabolic Activity: Effects on Serum Concentrations of Aripiprazole, Haloperidol, Risperidone, Paliperidone and Zuclopenthixol. *Eur J Clin Pharmacol* **2016**, *72*, 175–184, doi:10.1007/s00228-015-1965-1.
29. Scherf-Clavel, M.; Frantz, A.; Eckert, A.; Weber, H.; Unterecker, S.; Deckert, J.; Reif, A.; Hahn, M. Effect of CYP2D6 Pharmacogenetic Phenotype and Phenoconversion on Serum Concentrations of Antidepressants and Antipsychotics: A Retrospective Cohort Study. *Int J Clin Pharm* **2023**, *45*, 1107–1117, doi:10.1007/s11096-023-01588-8.
30. Tonti, E.; Lee, Y.M.; Gruenke, N.; Ferren, J.; Stutzman, D.L. Impact of Pharmacogenomics on Pediatric Psychotropic Medication Prescribing in an Ambulatory Care Setting. *Journal of Child and Adolescent Psychopharmacology* **2024**, *34*, 52–60, doi:10.1089/cap.2023.0087.
31. Gerlach, S.; Maruf, A.A.; Shaheen, S.M.; McCloud, R.; Heintz, M.; McAusland, L.; Arnold, P.D.; Bousman, C.A. Prevalence Estimates of Cytochrome P450 Phenoconversion in Youth Receiving Pharmacotherapy for Mental Health Conditions. *Clin Pharma and Therapeutics* **2025**, *117*, 670–675, doi:10.1002/cpt.3534.
32. Gerlach, S.; Maruf, A.A.; Shaheen, S.M.; McCloud, R.; Heintz, M.; McAusland, L.; Arnold, P.D.; Bousman, C.A. Effect of CYP2D6 Genetic Variation on Patient-Reported Symptom Improvement and Side Effects among Children and Adolescents Treated with Amphetamines. *Pharmacogenetics and Genomics* **2024**, *34*, 149–153, doi:10.1097/FPC.0000000000000529.
33. Jallaq, S.A.; Verba, M.; Strawn, J.R.; Martin, L.J.; DelBello, M.P.; Ramsey, L.B. CYP2D6 Phenotype Influences Aripiprazole Tolerability in Pediatric Patients with Mood Disorders. *Journal of Child and Adolescent Psychopharmacology* **2021**, *31*, 56–62, doi:10.1089/cap.2020.0058.
34. Lesche, D.; Mostafa, S.; Everall, I.; Pantelis, C.; Bousman, C.A. Impact of CYP1A2, CYP2C19, and CYP2D6 Genotype- and Phenoconversion-Predicted Enzyme Activity on Clozapine Exposure and Symptom Severity. *Pharmacogenomics J* **2020**, *20*, 192–201, doi:10.1038/s41397-019-0108-y.

35. Mostafa, S.; Polasek, T.M.; Sheffield, L.J.; Huppert, D.; Kirkpatrick, C.M.J. Quantifying the Impact of Phenoconversion on Medications With Actionable Pharmacogenomic Guideline Recommendations in an Acute Aged Persons Mental Health Setting. *Front. Psychiatry* **2021**, *12*, 724170, doi:10.3389/fpsyt.2021.724170.
36. Kiss, Á.; Menus, Á.; Tóth, K.; Déri, M.; Sirok, D.; Gabri, E.; Belic, A.; Csukly, G.; Bitter, I.; Monostory, K. Phenoconversion of CYP2D6 by Inhibitors Modifies Aripiprazole Exposure. *Eur Arch Psychiatry Clin Neurosci* **2020**, *270*, 71–82, doi:10.1007/s00406-018-0975-2.
37. Zourkova, A.; Jurica, J.; Hadasova, E.M.; Pulkrabkova, L. P.2.c.008 Fading of the Inhibition of CYP2D6 after Paroxetine Treatment Discontinuation. *European Neuropsychopharmacology* **2008**, *18*, S328–S329, doi:10.1016/S0924-977X(08)70454-1.
38. Mostafa, S.; Kirkpatrick, C.M.J.; Byron, K.; Sheffield, L. An Analysis of Allele, Genotype and Phenotype Frequencies, Actionable Pharmacogenomic (PGx) Variants and Phenoconversion in 5408 Australian Patients Genotyped for CYP2D6, CYP2C19, CYP2C9 and VKORC1 Genes. *J Neural Transm* **2019**, *126*, 5–18, doi:10.1007/s00702-018-1922-0.
39. Ishtiak-Ahmed, K.; Lunenburg, C.; Thirstrup, J.; Clausen, L.; Thomsen, P.; Gasse, C. Pharmacogenetic CYP2D6 Variability, Phenoconversion and Treatment Outcomes: A Danish Population-Based Cohort Study in 6,798 Individuals Initiating Atomoxetine Treatment. *European Psychiatry* **2022**, *65*, S227–S227, doi:10.1192/j.eurpsy.2022.588.
40. Gill, P.S.; Elchynski, A.L.; Porter-Gill, P.A.; Goodson, B.G.; Scott, M.A.; Lipinski, D.; Seay, A.; Kehn, C.; Balmakund, T.; Schaefer, G.B. Multidisciplinary Consulting Team for Complicated Cases of Neurodevelopmental and Neurobehavioral Disorders: Assessing the Opportunities and Challenges of Integrating Pharmacogenomics into a Team Setting. *Journal of Personalized Medicine* **2022**, *12*, 599, doi:10.3390/jpm12040599.
41. Berm, E.; Kok, R.; Hak, E.; Wilffert, B. Relation between CYP2D6 Genotype, Phenotype and Therapeutic Drug Concentrations among Nortriptyline and Venlafaxine Users in Old Age Psychiatry. *Pharmacopsychiatry* **2016**, *49*, 186–190, doi:10.1055/s-0042-105443.
42. Fan, M.; Xue, Z.; Huafang, L.; Shunying, Y. Phenotypic Transformation of Drug-Metabolizing Enzymes and Psychotropic Drug Therapy. **2022**, *55*, 145–149, doi:10.3760/cma.j.cn113661-20210623-00201.
43. Shilbayeh, S.A.R.; Adeen, I.S.; Alhazmi, A.S.; Ibrahim, S.F.; Al Enazi, F.A.R.; Ghanem, E.H.; Binduraihem, A.M. The Frequency of CYP2D6 and CYP3A4/5 Genotypes and The Impact of Their Allele Translation and Phenoconversion-Predicted Enzyme Activity on Risperidone Pharmacokinetics in Saudi Children with Autism. *Biochem Genet* **2024**, *62*, 2907–2932, doi:10.1007/s10528-023-10580-w.
44. Eap, *Chin B; Ranjbar, S.; Gamma, F.; Von Plessen, K.; Von Gunten, A.; Conus, P.; Gras, C.; Piras, M. CYP2D6 GENOTYPES AND TREATMENT FAILURE WITH RISPERIDONE OR ARIPIPRAZOLE: RESULTS FROM A ONE-YEAR LONGITUDINAL STUDY. *Int J Neuropsychopharmacol* **2025**, *28*, i88–i89, doi:10.1093/ijnp/pyae059.152.
45. Deutsche Gesellschaft Für Experimentelle Und Klinische Pharmakologie Und Toxikologie e.V. Abstracts of the 81th Annual Meeting, March 10-12, 2015, Kiel, Germany. *Naunyn-Schmiedeberg's Arch Pharmacol* **2015**, *388*, 1–98, doi:10.1007/s00210-015-1087-4.
46. Magalhães, P.; Alves, G.; Fortuna, A.; Llerena, A.; Falcão, A. Real-World Clinical Characterization of Subjects with Depression Treated with Antidepressant Drugs Focused on (Non-)Genetic Factors, Pharmacokinetics, and Clinical Outcomes: GnG-PK/PD-AD Study. *Experimental and Clinical Psychopharmacology* **2020**, *28*, 202–215, doi:10.1037/pha0000294.
47. Russell, J.; Arwood, M.J.; Toro-Pagán, N.M.D.; Amin, N.S.; Cambridge, M.D.; Turgeon, J.; Michaud, V. Case Report: Performing a Medication Safety Review Assisted by Pharmacogenomics to Explain a Prescribing Cascade Resulting in a Patient Fall. *Medicina* **2023**, *59*, 118, doi:10.3390/medicina59010118.
48. Jessurun, N.T.; Vermeulen Windsant, A.; Mikes, O.; Van Puijenbroek, E.P.; Van Marum, R.J.; Grootens, K.; Derijks, H.J. Inhibition of CYP2D6 with Low Dose (5 Mg) Paroxetine in Patients with High 10-hydroxynortriptyline Serum levels-A Prospective Pharmacokinetic Study. *Brit J Clinical Pharma* **2021**, *87*, 1529–1532, doi:10.1111/bcp.14455.

49. Hemeryck, A.; Lefebvre, R.; Devriendt, C.; Belpaire, F. Paroxetine Affects Metoprolol Pharmacokinetics and Pharmacodynamics in Healthy Volunteers. *Clinical Pharmacology & Therapeutics* **2000**, *67*, 283–291, doi:10.1067/mcp.2000.104788.
50. Parker, R.B.; Soberman, J.E. Effects of Paroxetine on the Pharmacokinetics and Pharmacodynamics of Immediate-Release and Extended-Release Metoprolol. *Pharmacotherapy* **2011**, *31*, 630–641, doi:10.1592/phco.31.7.630.
51. Goryachkina, K.; Burbello, A.; Boldueva, S.; Babak, S.; Bergman, U.; Bertilsson, L. Inhibition of Metoprolol Metabolism and Potentiation of Its Effects by Paroxetine in Routinely Treated Patients with Acute Myocardial Infarction (AMI). *Eur J Clin Pharmacol* **2008**, *64*, 275–282, doi:10.1007/s00228-007-0404-3.
52. Damborská, A.; Hanáková, L.; Pindurová, E.; Horská, K. Case Report: Therapeutic Drug Monitoring and CYP2D6 Phenoconversion in a Protracted Paroxetine Intoxication. *Front. Pharmacol.* **2024**, *15*, 1444857, doi:10.3389/fphar.2024.1444857.
53. Del Toro-Pagán, N.M.; Matos, A.; Bardolia, C.; Michaud, V.; Turgeon, J.; Amin, N.S. Pharmacist Assessment of Drug-Gene Interactions and Drug-Induced Phenoconversion in Major Depressive Disorder: A Case Report. *BMC Psychiatry* **2022**, *22*, 46, doi:10.1186/s12888-021-03659-4.
54. Del Toro-Pagán, N.M.; Matos, A.; Thacker, D.; Turgeon, J.; Amin, N.S.; Michaud, V. Pharmacist-Led Medication Evaluation Considering Pharmacogenomics and Drug-Induced Phenoconversion in the Treatment of Multiple Comorbidities: A Case Report. *Medicina* **2021**, *57*, 955, doi:10.3390/medicina57090955.
55. Michaud, V.; Dow, P.; Turgeon, J. Illustrative and Historic Cases of Phenoconversion.
56. Nahid, N.A.; McDonough, C.W.; Wei, Y.-J.J.; Gong, Y.; Empey, P.E.; Haddad, A.; Fillingim, R.B.; Johnson, J.A. *CYP2D6* Phenotypes and Emergency Department Visits Among Patients Receiving Opioid Treatment. *JAMA Netw Open* **2025**, *8*, e2523543, doi:10.1001/jamanetworkopen.2025.23543.
57. Fulton, C.R.; Zang, Y.; Desta, Z.; Rosenman, M.B.; Holmes, A.M.; Decker, B.S.; Zhang, Y.; T Callaghan, J.; Pratt, V.M.; Levy, K.D.; et al. Drug–Gene and Drug–Drug Interactions Associated with Tramadol and Codeine Therapy in the INGENIOUS Trial. *Pharmacogenomics* **2019**, *20*, 397–408, doi:10.2217/pgs-2018-0205.
58. Muhn, S.; Amin, N.S.; Bardolia, C.; Toro-Pagán, N.D.; Pizzolato, K.; Thacker, D.; Turgeon, J.; Tomaino, C.; Michaud, V. Pharmacogenomics and Drug-Induced Phenoconversion Informed Medication Safety Review in the Management of Pain Control and Quality of Life: A Case Report. *Journal of Personalized Medicine* **2022**, *12*, 974, doi:10.3390/jpm12060974.
59. Kummer, O.; Hammann, F.; Moser, C.; Schaller, O.; Drewe, J.; Krähenbühl, S. Effect of the Inhibition of CYP3A4 or CYP2D6 on the Pharmacokinetics and Pharmacodynamics of Oxycodone. *Eur J Clin Pharmacol* **2011**, *67*, 63–71, doi:10.1007/s00228-010-0893-3.
